# A spectral framework to map QTLs affecting joint differential networks of gene co-expression

**DOI:** 10.1371/journal.pcbi.1012953

**Published:** 2025-04-17

**Authors:** Jiaxin Hu, Jesse N. Weber, Lauren E. Fuess, Natalie C. Steinel, Daniel I. Bolnick, Miaoyan Wang

**Affiliations:** 1 Department of Statistics, University of Wisconsin-Madison, Madison, Wisconsin, United States of America; 2 Department of Integrative Biology, University of Wisconsin-Madison, Madison, Wisconsin, United States of America; 3 Department of Biology, Texas State University, San Marcos, Texas, United States of America; 4 Department of Biological Sciences, University of Massachusetts Lowell, Lowell, Massachusetts, United States of America; 5 Department of Ecology and Evolutionary Biology, University of Connecticut, Storrs, Connecticut, United States of America; University of North Texas, UNITED STATES OF AMERICA

## Abstract

Studying the mechanisms underlying the genotype-phenotype association is crucial in genetics. Gene expression studies have deepened our understanding of the genotype  →  expression  →  phenotype mechanisms. However, traditional expression quantitative trait loci (eQTL) methods often overlook the critical role of gene co-expression networks in translating genotype into phenotype. This gap highlights the need for more powerful statistical methods to analyze genotype  →  network  →  phenotype mechanism. Here, we develop a network-based method, called spectral network quantitative trait loci analysis (snQTL), to map quantitative trait loci affecting gene co-expression networks. Our approach tests the association between genotypes and joint differential networks of gene co-expression via a tensor-based spectral statistics, thereby overcoming the ubiquitous multiple testing challenges in existing methods. We demonstrate the effectiveness of snQTL in the analysis of three-spined stickleback (*Gasterosteus aculeatus*) data. Compared to conventional methods, our method snQTL uncovers chromosomal regions affecting gene co-expression networks, including one strong candidate gene that would have been missed by traditional eQTL analyses. Our framework suggests the limitation of current approaches and offers a powerful network-based tool for functional loci discoveries.

## Author summary

Our work addresses a key gap in understanding the mechanistic foundations for genotype-phenotype associations. While existing expression quantitative trait loci (eQTL) methods identify candidate loci affecting gene expression variants, they often neglect the crucial role of gene co-expression networks. Here, we develop a network-based QTL framework to map genetic loci affecting the gene co-expression network. Utilizing a tensor-based spectral approach, our snQTL method estimates the differential co-expression patterns and effectively identifies the associated genetic loci. Application of snQTL to three-spined sticklebacks revealed candidate loci missed by standard methods and estimated network changes led by genetic variants. Our result suggests the limitations of current approaches and highlights the potential of network-based functional loci discovery. Our method presents a novel solution for comparing biological networks, opening doors for deeper network analysis in genetics.

## Introduction

The identification of genetic variants underlying complex phenotypic traits has been a pivotal area in genetics research for decades. Genome-wide association studies (GWASs) have identified important genetic variants by detecting statistical association between phenotypes and genotypes in outbred populations [38]. Likewise, quantitative trait locus (QTL) mapping in experimentally crossbred organisms allows researchers to shuﬄe genetic backgrounds meiotically and test for associations between measurable phenotypes and chromosomal regions. However, both GWAS and QTL mapping are limited by the challenge of elucidating the mechanisms behind these genotype-phenotype associations, and the lack of sufficient functional information for many loci [6,29]. Gene expression studies can bridge this gap between genotype and phenotype. To this end, expression quantitative trait locus (eQTL) analysis was developed to identify associations between genetic variants and gene expression levels [7]. The eQTL studies have deepened our understanding of genotype  →  expression  →  phenotype mechanisms [28,29,39,44]. Existing eQTL methods have identified numerous genetic loci, categorized as cis- or trans-eQTL, that influence gene expression. Cis-eQTLs are located near the expressed gene on the same chromosome and influence gene expression by either affecting the binding of transcription factors or chromatin proteins to DNA [41,45] or being in linkage disequilibrium (LD) with such regulatory elements. Growing evidence has indicated that cis-eQTLs do not fulfill the expected role of linking genetic variants to target genes or pathways [54–58]. Conversely, trans-eQTLs are typically located far from the expressed gene on the same chromosome or on different chromosomes.

A key limitation of current eQTL studies is their focus on individual genes, but not on the network structure of gene co-expression. Gene co-expression networks are often represented by correlation matrices at the whole-transcriptome scale [30,31,51]. Correlation among gene expressions may arise, for example, when multiple genes are co-regulated by the same transcription factor or participate in sequential regulatory cascades. Correlated expression can also arise from genetic linkage between separate regulatory cascades and from shared environmental effects, although in this case correlated expression do not necessarily imply direct functional interactions.

There is accumulating evidence that gene co-expression networks can differ between species [32,37] or populations [32], even in controlled environments. These differences suggest the gene co-expression network is evolvable, and hence most likely has a genetic basis. The co-expression might evolve with genetic variants, for instance, if transcription co-factor A modifies the effect of transcription factor B on target gene C’s expression, then allelic differences at gene A can modify the correlation between expression of genes B and C. Mutations that alter gene linkage patterns (e.g., inversions or translocations) could also alter gene co-expression networks. This concept is similar to mapping epistatic eQTLs [12], except that those studies (excluding work on highly prolific laboratory models) only rarely have the power to identify more than a few interacting genes. If genetic variants broadly alter co-expression network structure, eQTL or GWAS methods could in principle map these genetic loci. Analyzing these associations between genetic loci and co-expression network can reveal the network-level impact of quantitative trait loci, leading to new insights into the genetic basis of complex traits. Developing efficient methods for network-based eQTL is a topic of great interest.

Recent studies have extended the concept of eQTL to co-QTLs [6,8,9,11]. These methods aim to identify genetic loci that explain coordinated changes in expression between pairs of genes. However, current co-QTL methods have several limitations. One of the challenges is the massive number of statistical tests needed, which increases quadratically with the number of gene pairs analyzed. Some methods restrict co-QTL searches to previously identified eQTLs [8,9], while others prioritize gene pairs based on prior knowledge [6]. These approaches reduce testing burdens but may miss important co-QTLs. Furthermore, current co-QTL methods are limited by assuming linear models with additive effects [6,8,9,11]. The additive assumption neglects dominance, recessiveness, or even transgressive inheritance, hindering the ability to capture the full genetic influence on co-expression networks. Recent work on activity QTL (aQTL) [56] has extended traditional eQTL analysis by using gene activity scores inferred from co-expression networks. However, this method fails to accommodate multiple networks or relax the linear assumptions in co-QTL analysis. More powerful network-based eQTL methods are needed to address these issues.

In this paper, we propose a novel method called spectral network QTL (snQTL) to address these challenges. Our snQTL approach identifies the association between genotype and the entire co-expression network structure. The identified loci, which we also refer to as snQTLs, explain a fraction of the genetic variance of the entire gene co-expression network. The snQTL represent genetic variants that alter the global pattern of a network, while traditional co-QTLs represent genetic variants that alter the expression for only a particular pair of genes. Statistically, the key idea of the snQTL method is to use tensor spectral statistics to represent the joint difference in gene co-expression networks at each of many different loci. This approach reduces the number of tests to the number of genetic markers throughout the genome of a recombinant hybrid population (used for mapping), and we allow for the simultaneous consideration of all active genes in the network. We also propose a permutation-based approach to obtain valid testing results that are robust to the data distribution. In addition to identifying snQTLs, our snQTL framework also outputs the joint differential networks, which represents the specific network patterns that are altered by genetic variants at the detected snQTLs. Our approach has the potential to be extended to mapping genetic effects on the architecture of microbiome co-occurrence networks and proteomic networks. We demonstrate the effectiveness of our method in the immune tissue gene expression data from a large genetic cross of three-spined stickleback fish (*Gasterosteus aculeatus*).

## Results

### Spectral network QTL framework

**Fig 1:**
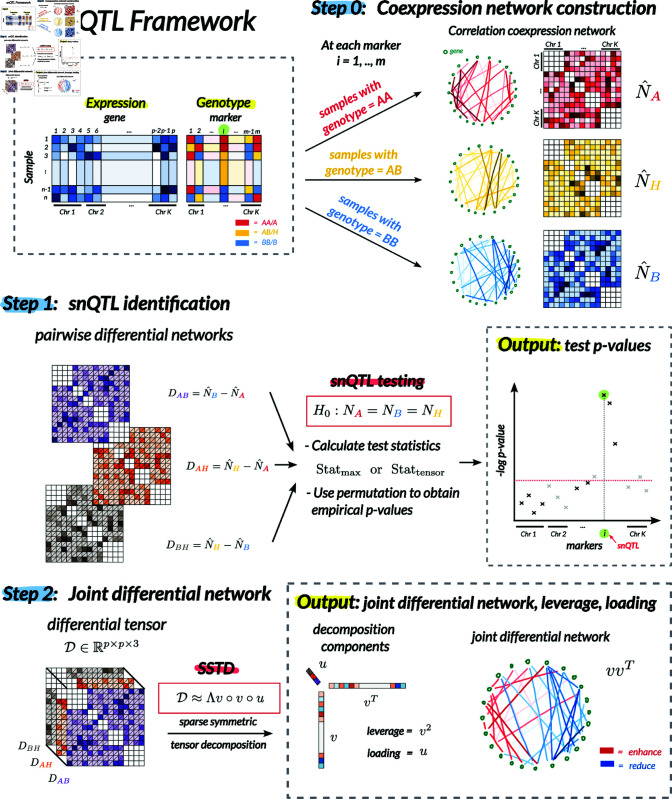
The main idea of our snQTL framework. Our snQTL framework takes as input (i) gene expression read counts and (ii) genotypes of genetic markers from the same set of samples. The snQTL approach consists of three steps: (0) co-expression network construction, (1) snQTL identification via hypothesis testing using multilinear spectral statistics, and (2) joint differential network estimation at associated loci via sparse symmetric tensor decomposition. At each marker, the output includes (i) a p-value indicating the association significance between the co-expression network and the marker, and (ii) a joint differential network with nodes representing genes and edges representing associated effects.

Fig 1 illustrates the main framework of our snQTL method. We take as input (i) expression read counts of *p* genes and (ii) genotypes of *m* genetic markers, from the same set of *n* individuals. The snQTL method then outputs two key results at each marker: (i) a p-value indicating the association significance between the co-expression network and the marker, and (ii) a joint differential network with nodes representing genes and edges representing associated effects.

The snQTL consists of three steps. First, we construct gene co-expression networks; see Step 0 in Fig 1. At each of the *m* markers, we group the samples by genotype (AA, AB, BB). For each group, we calculate a Pearson correlation matrix using only the gene expression data within that group. Let $N_{A},N_{B},N_{H}$ denote the (unknown) population correlation matrices, where *A* and *B* denote homozygous genotypes and *H* denotes heterozygous genotype. We exclude within-chromosome correlations by setting the (*j*,*k*)-th entries in $N_{A},N_{B},N_{H}$ to zero, if genes *j* and *k* are located on the same chromosome. The purpose of this ‘set-to-zero’ step is to focus on trans-snQTLs that affect between-chromosome co-expressions which are less likely to result from LD and more likely to indicate functional connections. In eQTL studies, truly functional trans-eQTLs often influence the expression or structure of transcription factors, ultimately affecting their ability to regulate distant genes [40,46,47]. Although, in theory, a trans-eQTL could act on a nearby gene (e.g., if a transcription factor regulates a gene immediately adjacent on a chromosome), QTL mapping often cannot distinguish cis-eQTLs from trans-eQTLs on the same chromosome arm. For this reason, in this paper we use an operational definition of trans-eQTLs as those acting across chromosomes, where they can be confidently distinguished. If desired, the ‘set-to-zero’ step can be skipped to include within-chromosome correlations in the snQTL analysis.

Next, we perform statistical tests to identify genetic markers affecting co-expression networks; see Step 1 in Fig 1. At each marker *i*, we test the null hypothesis:


$$\begin{array}{ccc} H_{0}:N_{A}=N_{B}=N_{H}. & & \end{array}$$ (1)

In the next section, we will provide several test statistics based on the sparse multilinear spectral components of the correlation matrices. Let $D_{AB}={\hat{N}}_{B}\;-\;{\hat{N}}_{A}$, $D_{AH}={\hat{N}}_{H}\;-\;{\hat{N}}_{A}$, and $D_{BH}={\hat{N}}_{H}\;-\;{\hat{N}}_{B}$ denote the three pairwise differential networks, where $\hat{\cdot }$ denotes the sample correlation matrix. The sparse multilinear spectral components of differential networks allow us to test for classical genetic dominance effects as well as a broad range of genetic effects onto the entire co-expression networks. We use permutation to obtain the *p*-value for the hypothesis test in Eq (1). The output is summarized as a Manhattan plot of association *p*-values across the genome. Our framework allows both traditional cases where the sample size is greater than the number of genes (*n*>*p*) and to high-dimensional cases where *n*<*p*. We address high-dimensionality by introducing sparsity into the test statistics and using permutation testing for a robust assessment of the null distribution.

Last, we estimate the joint differential network at the associated marker; see Step 2 in Fig 1. The joint differential network is a gene network with weighted edges that represent co-expressions affected by a genetic marker. Unlike the pairwise differential network, which describes the co-expression changes just between two genotypes (e.g., AA versus AB), the joint differential network summarizes prominent co-expression changes across three genotypes. We use sparse tensor decomposition to obtain the leading eigenvectors in the pairwise differential correlations. These eigenvectors summarize the differential signal into a single network. The resulting joint differential network has nodes representing genes, edges representing co-expression changes associated with the genetic marker, and edge weights and signs representing the magnitude and direction (enhancement or reduction) of these changes.

#### snQTL testing and joint differential network estimation via sparse tensordecomposition

We briefly introduce the sparse symmetric tensor decomposition (SSTD) in our contexts. Let $D\in {\mathbb{R}}^{p\times p\times q}$ be an order-3 tensor with each of the *q* slides being a symmetric *p*-by-*p* matrix. We say *D* is sparse and of rank 1 if *D* satisfies the SSTD model:


$$D=\Lambda v\circ v\circ u,\text{ or equivalently }D_{jkl}=\Lambda v_{j}v_{k}u_{l},$$

for all  ( *j* , *k* , *l* ) ∈ { 1 , … , *p* } × { 1 , … , *p* } × { 1 , … , *q* } , where  ∘  denotes the vector outer product, *v* and *u* are norm-1 vectors in ${\mathbb{R}}^{p}$ and ${\mathbb{R}}^{q}$, respectively, and *v* is further sparse with $∥v{∥}_{0}\leq R$ for some constant *R* ≤ *p*, and $\Lambda \in {\mathbb{R}}_{+}$. Here $∥\;\cdot \;{∥}_{0}$ is the *L*_0_ norm that counts the number of non-zero entries in the vector. The constraint on $∥v{∥}_{0}$ controls the sparsity on the first two modes, with a smaller *R* resulting in a sparser *v*. We call *Λ*, *v*, and *u*, the sparse leading tensor eigenvalue (sLTE), the sparse tensor eigenvector, and the loading vector, respectively.

In our snQTL framework, we define an order-3 differential tensor $D\in {\mathbb{R}}^{p\times p\times 3}$ by stacking the three pairwise differential networks $D_{AB},D_{AH},D_{BH}$ together. To summarize the signal in *D*, we compute the SSTD approximation to the tensor *D*. Specifically, we solve for the spectral components  ( *Λ* , *v* , *u* )  that minimize the least square approximation error


$$\begin{array}{ccc} \underset{\begin{array}{c} (\Lambda ,v,u)\in {\mathbb{R}}_{+}\times {\mathbb{R}}^{p}\times {\mathbb{R}}^{q},\\ ∥v{∥}_{2}=∥u{∥}_{2}=1,∥v{∥}_{0}\leq R \end{array}}{\min}∥D-\Lambda v\circ v\circ u{∥}_{F}^{2}, & & \end{array}$$ (2)

where $∥\;\cdot \;{∥}_{F}$ denotes the Frobenius norm defined as the squared sum of tensor entries, and $∥\;\cdot \;{∥}_{2}$ denotes the vector *L*_2_ norm. We denote the sLTE solution as *Λ* ( *D* ) , with *D* being the input differential tensor. The sLTE *Λ* ( *D* )  represents the global strength of the co-expression changes across the three genotype groups. A larger sLTE suggests a stronger deviation from the null hypothesis in (1). Our test statistics, named ${\text{Stat}}_{\text{test}}$, is defined using the sLTE:


$$\begin{array}{ccc} {\text{Stat}}_{\text{tensor}}=\Lambda (D). & & \end{array}$$ (3)

Our snQTL also features the estimation of a joint differential network. The sparse tensor eigenvector, *v* = *v* ( *D* ) , and loading vector, *u* = *u* ( *D* ) , together capture a lower-dimensional representation of *D*. We call the leading matrix approximation, ${vv}^{T}$, the “joint differential network". This network captures the overall co-expression network changes in response to the genetic variation at the marker of interest. We call the element-wise squared eigenvector, denoted as $v^{2}$, the “gene leverage". The vector *v* reflects the contribution of genes to the differential network, with higher values indicating greater connectivity within the network. The loading vector, *u*, represents the weights for comparisons among the three genotype groups (e.g., AA-AB, AA-BB, AB-BB), with larger magnitudes indicating a greater contribution from each comparison.

Our snQTL is inspired from earlier work on SSTD [16]. However, we introduce key modifications that tailor SSTD to our specific needs in snQTL analysis. We explicitly considers the symmetry and sparsity in the first two modes of the tensor, making SSTD a better fit for our framework (details in *Materials and methods*). Furthermore, unlike earlier work that focuses on the decomposition only [16], our primary goal is hypothesis testing within the context of snQTL analysis. We have developed specific tools for this purpose.

#### snQTL testing via sparse matrix decomposition

We also propose an optional statistic for (1), based on extension of sparse leading matrix eigenvalue (sLME) [13]. The sLME of a matrix *D* is defined as


λ(D)= max ⁡ v∈ℝp,∥v∥2=1,∥v∥0≤R|vTDv|. (4)

The sLME represents the maximum eigenvalue of matrix *D* subject to the sparse eigenvectors. Our second test statistics, named “max", is defined as the maximal sLME from all three pairwise comparisons:


$${\text{Stat}}_{\text{max}}=\max\{\lambda (D_{AB}),\;\lambda (D_{AH}),\;\lambda (D_{BH})\}.$$

Under the null hypothesis in (1), all pairwise differences ($D_{AB},D_{AH},D_{BH}$) are zero matrices, resulting in a zero max statistic. Conversely, a larger max statistic indicates higher differences in at least one pairwise comparison, making it well-suited for joint comparison of multiple networks.

Our max statistic generalizes the earlier work from pairwise comparison [13] to joint comparison of multiple matrices. Other methods include $L_{2}$-type statistics [14] that consider all entries in the comparison, and $L_{\infty }$-type statistics [15] that focus on the largest deviation. However, the $L_{2}$-type statistics assume all genes contribute equally, while the $L_{\infty }$-type statistics capture only the single most extreme gene pair. In contrast, the spectral statistic, sLME, is well-suited for scenarios where the genetic effects are weak and sparse, meaning that a small subset of genes exhibit moderate effects [13,52,53]. This aligns with the biological expectation that genes might have significant but subtle co-expression changes. Additionally, the sparsity in sLME promotes result interpretability and faster computation.

#### Algorithm implementation

We design an iterative algorithm that alternatively updates the decomposition components to approximately solve (2). We adopt the penalized matrix decomposition [13,19] to approximately solve for sLME in (4). In practice, we also consider variants of tensor and max statistics, such as the sum of sLMEs and the squared sLTE (*S1 Appendix*). More variants can be designed based on problem contexts. If the joint differential network is of interest, the tensor statistic is recommended, as the tensor approach facilitates network estimation. If the goal is solely snQTL testing, both tensor and matrix approaches perform similarly. For all test statistics, we use permutation to approximate the null distributions and obtain the empirical p-values. The number of permutations and the sparsity hyper-parameter *R* can be adjusted as needed. See *Materials and methods* for more details. The code implementation of snQTL is provided at https://github.com/Marchhu36/snQTL.

#### Analysis of simulated data

In the simulation, we focus on the individuals drawn from an F2 hybrid generation derived from crosses between genetically divergent populations, with parent-of-origin diagnostic genetic markers spread across all chromosomes. This is to mimic the genotype patterns in the three-spined stickleback data. We also performed similar experiment using GWAS synthetic data; the simulation results are in the “*S1 Appendix*”. In general, our approach is suitable for any other type of cross designs.

We first evaluated the efficiency of our snQTL framework on synthetic data for 200 genes across 20 chromosomes. We started with genetically divergent homozygous parents, and simulated the genotypes for an F1 cross and for an F2 intercross generation with random chromosomal crossing overs. For each F1 gamete, we simulated one recombination event per chromosome per gamete, randomly placed along the chromosome with a uniform distribution. The F2 hybrids’ gene expression counts were generated from Poisson distributions with parameters varying by genotypes. We randomly selected one gene as the snQTL and generated three network effects associated with the snQTL. Then, we altered the expression levels of all 200 genes based on the additive network effect associated with this selected snQTL. In our current simulations, all 200 genes were considered candidate loci for snQTL detection. In real scenarios, any marker linked to genetic variants – such as single nucleotide polymorphism (SNPs), indels, or haplotypes – could serve as candidate loci for snQTL detection. We tested the framework with varying hybrid population sizes from 50 to 500 to assess performance cover various scenarios. The detailed procedures for synthetic data generation are in *S1 Appendix*.

**Fig 2:**
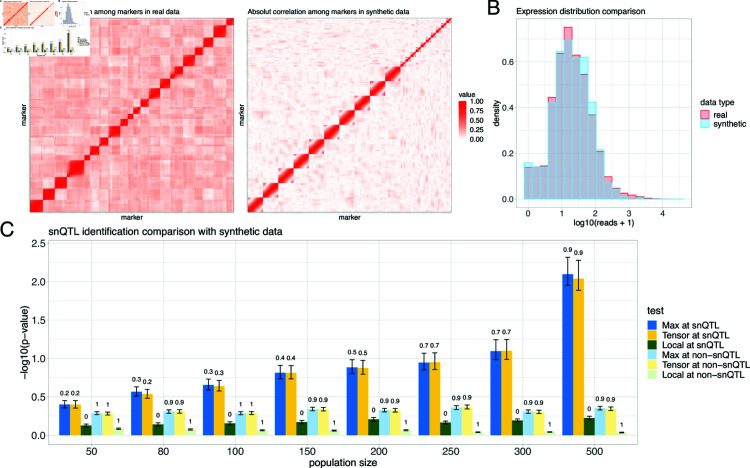
Analysis of simulated data. Synthetic datasets in three panels have the same parameter setup. (A) Absolute genetic correlation heatmaps among the markers in real F2 hybrid three-spined stickleback data [10] and synthetic data. Markers are ordered following their positions on the genome. Genetic correlations are measured by absolute sample Pearson correlation coefficients between the genotypes of two markers. (B) Density histograms for expression counts in real stickleback and synthetic data. The parameters in synthetic data generation are carefully chosen to mimic the real data. (C) Barplots comparing the snQTL identification performances for snQTL framework and local method (F-test for regression of pairwise co-expression onto genotype) on synthetic data with varying population size from 50 to 500. We set sparsity parameter *R* = *p* in snQTL for a fair comparison with the non-sparse local method. For results labeled “at snQTL", the y-axis is the observed $-log_{10}(\text{p-value})$ for tests at the single true snQTL; for results labeled “at non-snQTL", the y-axis is the averaged observed $-log_{10}(\text{p-value})$ from three tests at randomly selected non-snQTL markers. True positive (or negative) rates for the tests at snQTL (or non-snQTL) are shown above the bars. All reported numbers are averaged across 50 replications for each population size.

Fig 2 confirms the similarity between the synthetic and real F2 hybrid three-spined stickleback data [10]. The similar block diagonal patterns in the genetic correlation heatmaps (Fig 2A) suggest the LD among real and simulated markers. In both real and synthetic data, markers from the same chromosome show higher genotype correlations than those from different chromosomes. Our synthetic genotype generation captures this notable block structure. The overlapped histograms of expression counts (Fig 2B) validate our simulation procedures, indicating parameter values effectively mimicked real datasets.

We compared three methods on the synthetic data: the snQTL framework with max statistic, with tensor statistic, and a local approach based on F-tests for linear regressions of pariwise co-expression against genotypes. This local approach is similar to previous co-QTL analyses [6,8]. We assessed both statistical power and type I error by applying all tests at the snQTL and non-snQTLs. Average test p-values and true positive (TP)/negative (TN) rates were recorded across 50 replicates for each population size.

Fig 2C demonstrates the superior statistical power of our snQTL framework, especially with larger populations. Our snQTL-based methods demonstrate a significant improvement as the sample size increases, while the local method shows only a minor improvement with additional samples. The out-performance suggests that the snQTL framework effectively addresses the multiple testing burden and tends to lead to more discoveries than the local approach. Additionally, the high TN rates at non-snQTLs support the high accuracy of the snQTL framework for snQTL identification. This outerperformance of snQTL is consistent across various simulation setups, including GWAS-like synthetic data, different sparsity parameters, and hybrid generations. For extra simulations, please refer to *S1 Appendix*.

### Performing snQTL to map stickleback loci affecting co-expression networks

We conducted snQTL analysis on the three-spined stickleback (*Gasterosteus aculeatus*) data [10] to reveal the genetic landscape for co-expression networks in sticklebacks. These datasets are from a QTL mapping study in which wild fish were obtained from two lakes on Vancouver Island (Roberts Lake and Gosling Lake; RR and GG), and eggs/sperm mixed in petri dishes to generate F1 hybrids (RG). These hybrids were reared to maturity in an aquarium lab at the University of Texas and intercrossed to generate F2 intercross hybrids (RG*RG) and reciprocal backcrosses (RG*GG, GG*RG, RG*RR, RR*RG). Although hybrid crosses constituted a mixture of maternal backgrounds, maternal effects were excluded in our analyses. All F2 generation fish were reared to maturity in the laboratory and experimentally exposed to a cestode parasite, then euthanized 42 days post-exposure. Transcriptomic dataset was collected from head kidneys (pronephros, a major immune organ in fish) using Tag-Seq [18]. The cross design, sequencing methods, and bioinformatics pipelines are described in depth in earlier work [10,17].

The raw dataset consists of gene transcript counts and genotypes for 234 markers, for 351 samples from F2 generations and backcrosses. The genetic markers in the stickleback data are biallelic SNPs obtained from ddRADseq (reduced representation genomic sequencing) of the parental stickleback, and the hybrids. The SNPs identified by ddRADseq were filtered to only include ancestry-informative markers that exhibit fixed differences between the two populations (e.g., one allele is only found in Roberts Lake, the alternate allele is only found in Gosling Lake). Details of the genotyping procedure and SNP calling for mapping are provided in [10]. We preprocessed the data with the following procedure. First, to eliminate non-functional variations, we normalized the read count matrix and regressed expressions against the sex and population covariates, retaining the residuals (*S1 Appendix*). Second, we focused the analysis on the top 10,000 genes with the highest adjusted mean expressions, as more information may be involved with actively highly expressed genes. The number 10,000 was chosen to ensure computational efficiency. In general, our method allows more genes in the initial co-expression network analysis, since our method avoids multiple testing issues. Relaxing the filtering step could reveal important biological patterns, as it is unclear what expression levels lead to meaningful shifts in networks or phenotypes. Other filtering strategies, such as variance filtering and leveraging biological knowledge, may be helpful in other applications.

In addition, we considered the infection status of the sample fish as cestode infection is likely an environmental confounder. We added the worm presence as a predictor in the pre-processing regression step. Our snQTL analysis exhibited the same conclusions (*S1 Appendix*) before and after the additional procedure, suggesting the robustness of our discoveries to the infection status. For conciseness, we presented only the analysis without infection covariates in this paper. We leave further analyses involving more covariates and genes for future investigations.

#### Identification of stickleback snQTLs

**Fig 3:**
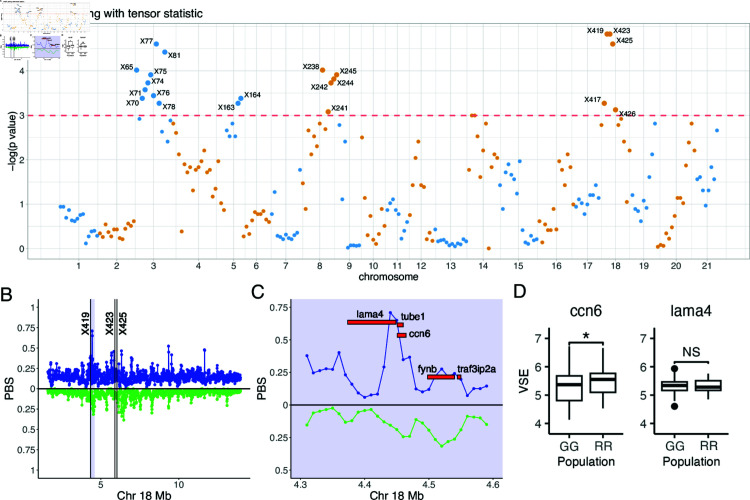
Identification of stickleback snQTLs via snQTL framework. (A) Manhattan plot for snQTL testing with tensor statistics marks 21 stickleback snQTLs, mainly clustered in Chr 3, Chr 8, and Chr 18. The y-axis represents the natural logarithms of p-values. The snQTLs are deemed with testing p-values smaller than 0.05 (above the dashed line). (B) Strong genomic targets of selection with high population branch statistic (PBS) distribute around the outstanding snQTLs (markers X419, X423, and X425) in Chr 18. Values above the medial line represent higher PBS in Gosling Lake (blue); values below the line represent higher PBS in Roberts Lake (green). (C) Zoomed-in shadowed area in (B). Development regulation genes, lama4 an d ccn6, locate tightly around marker X419 with high selection speed. (D) Variance stabilized expressions (VSE) for ccn6 and lama4 in Gosling (GG) and Roberts (RR) lakes.

We performed snQTL analysis on stickleback data using both tensor and max statistics. Both approaches lead to similar testing results (*S1 Appendix*), demonstrating the robustness of our snQTL identification. We present the findings using the tensor statistic here, as the tensor approach also facilitates joint differential network estimation. The Manhattan plot in Fig 3A shows 21 stickleback snQTLs concentrated at Chr 3, Chr 8, and Chr 18. This clustering pattern of snQTLs aligns with the LD structure among markers (Fig 2A). For instance, markers X77 and X75 exhibit highly correlated genotypes, leading both to be significant in snQTL tests. The three chromosomes of interest all exhibit extensive and stronger signals of snQTL associations compared to other chromosomes.

To further narrow down potential functional regions, we examined within each snQTL region for coding genes with strong genomic signatures of past natural selection. Specifically, we used published population genomic data: allele frequency estimates obtained from PoolSeq of  ∼  100 fish from each of three populations (Roberts Lake, Gosling Lake, and a marine outgroup). We calculated population branch statistics (PBS) measuring accelerated evolution in each lake (Roberts or Gosling), relative to an ancestral marine population (Sayward), as described in earlier work [10]. Large PBS in either lake population indicates a gene that was likely a target of natural selection within the lake in question, since its colonization  ∼  12,000 years ago.

**Fig 4:**
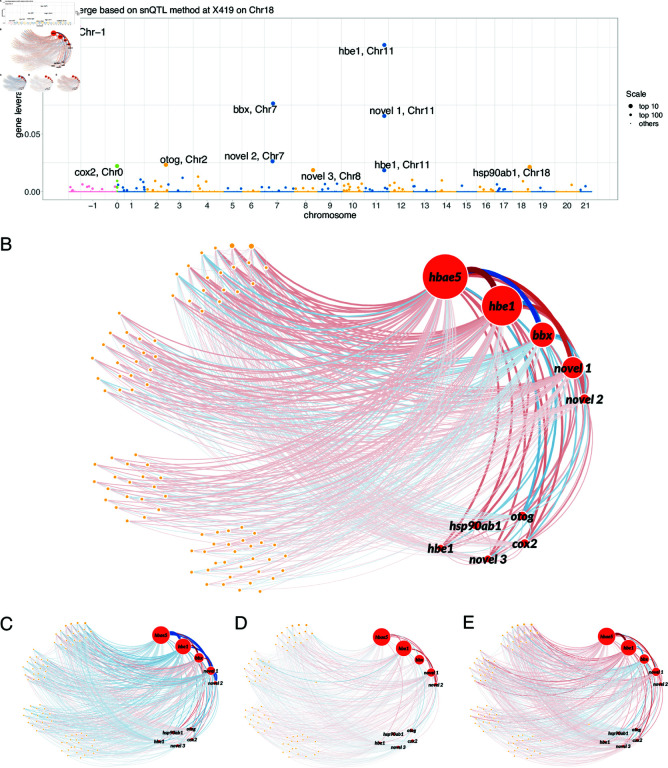
Joint differential network analysis at snQTL X419 on Chr 18. (A) Leverage scores for 10000 genes. Primary genes with top 10 leverage are highlighted with transcription IDs. Mitochondrial genome (MT) and scaffold region are coded as Chr 0 and Chr -1, respectively. (B-E) Networks for primary (red annotated nodes) and secondary (orange nodes) genes with top 100 leverages. The edge width indicates the connection strength between two genes; the diameter of node indicates the leverage of the gene; the color indicates enhancement (red) or reduction (blue) of the connection compared with average level. (B) Joint differential network at X419 with top 10% strongly connected edges. A wider edge implies a stronger genetic variation in the co-expression of the gene pair. Most genetic co-expression variations occur between the primary and secondary genes. (C-E) Co-expression networks corresponding to the genotypes GG, RG, and RR at X419, respectively. The linear changes in the colors of edges imply the nearly additive genetic effect to the co-expression networks. novel 1: ENSGACT00000018413; novel 2: ENSGACT00000026589; novel 3: ENSGACT00000017116.

Several protein-coding genes lie in regions adjacent to PBS outliers within snQTLs (*S1 Appendix*). We focused our analysis on genes near the largest snQTL on Chr 18 (Fig 3). None of these genes harbored coding variants but two were represented in our expression data: cellular communication network factor 6 (*ccn6*) and laminin subunity alpha 4 (*Lama4*). Although the *Lama4* expression differs little between parental populations, the *ccn6* expression was significantly lower in Gosling fish (*t* = 2 . 115 , *df* = 97 . 886 , *p* = 0 . 037, Fig 3D). In addition, *ccn6* is differentially expressed in our genetic crosses (*p* = 0 . 0511, *S1 Appendix*), but we found no evidence of protein coding changes between any of the populations. The gene *ccn6*, also known as *wisp3*, has 4 distinct protein domains that perform distinct functions [20], several of which have notable connections to the stickleback system. Secreted *ccn6* can bind to and limit insulin growth factor-1 (*igf-1*) signaling, thereby suppressing cell growth and metabolic potential [21], as well as mediating fibrotic responses [22,23]. The gene *ccn6* also acts as a transcription factor that activates genes necessary for formation of the mitochondrial electron transport system [24] and indirectly regulates reactive oxygen species (ROS) levels [25]. Gosling fish produce significantly less ROS, display less cestode-induced fibrosis, and grow faster than Roberts fish. It is worthy noting that in humans, the *ccn6* expression is largely restricted to kidney, skin and testes, consistent with an organ-specific regulatory role [10,26].

#### Joint differential network at snQTL locus X419

We further estimated joint differential networks for the significant snQTLs identified in our snQTL analysis. We found that most snQTLs are associated with similar sets of genes with high leverages, resulting in joint differential networks with comparable patterns (*S1 Appendix*). Here, we present the joint differential network at the most significant snQTL, X419 on Chr 18. We ranked genes based on their leverage scores from our method. We found that the top 10 genes achieved a cumulative leverage of 0.54, and the top 100 genes achieved a cumulative leverage of 0.9. We called the top 10 genes with highest leverages the “primary genes", and the remaining top genes the “secondary genes". These top 100 genes distribute widely on the genome, from the scaffold region and mitochondrial genome (MT) to all chromosomes (Fig 4A). This wide distribution of top genes implies the capacity of snQTLs to impact co-expressions throughout the whole genome. Such cross-chromosome influences are likely to represent functional genotype-network associations. None of the primary genes were identified in the previous differential expression (DE) analysis of pure parental populations [18], which highlights their unique roles in the co-expression network effects. In addition, the loading values for the genotype comparisons GG-RG and RG-RR are 0.498 and 0.31, respectively. The result suggests that the co-expressions between primary and secondary genes, except those with *bbx* and *otog*, are reduced in Gosling Lake fish and enhanced in Roberts Lake fish (Fig 4B). Moreover, the loading and co-expression networks for three genotypes (Fig 4C-E) show that differential networks for GG-RG and for RG-RR are comparable, indicating the nearly additive genetic effects to the co-expression networks.

We found that most genetic co-expression variations occur between a primary gene and a secondary gene and between two primary genes (Fig 4B). We note that many of the primary genes (*hbae5*, two *hbe1* paralogs, and the novel gene *ENSGACT00000018413*, which is orthologous to *hba2* in other species of fish) are hemoglobin subunits expressed in red blood cells and directly participate in oxygen transport activities, while the others are involved in closely related biological processes, such as blood vessel development (*hsp90ab1*) and carbohydrate metabolism (*otog*) (Table 1). These functions are consistent with decreased expression of *ccn6* being connected to elevated rates of *igf-1* signaling and cell replication in the head kidney, which is the hematopoietic organ in fish. Similarly, overexpression of heat shock proteins (i.e., *hsp90ab1*) can be stimulated either via pharmacological suppression of *igf-1* [27] or dysregulation of the electron transport chain in mitochondria, which is another major function of *ccn6*. Although the precise role of *mmp16b* has not been well characterized, *igf-1* is connected to the expression of other *mmp*s. Our analysis demonstrates the power of snQTL framework with functional annotation for unraveling the genetic basis of co-expression networks.

**Table 1:** List of primary genes with top 10 leverage scores in joint differential network at X419 on Chr 18.

Transcript ID	Leverage	Gene	Chr	Gene and Protein GO annotations
ENSGACT00000019169	0.1433	hbae5	Scaffold 112	heme, iron ion, oxygen binding; oxygen carrier activity
ENSGACT00000018425	0.1271	hbe1	11	heme, iron ion, oxygen binding; oxygen carrier activity
ENSGACT00000026622	0.0765	bbx	7	DNA binding
ENSGACT00000018413	0.0656	-	11	heme, iron ion, oxygen binding; oxygen carrier activity
ENSGACT00000026589	0.0263	-	7	uncharacterized
ENSGACT00000022959	0.0232	otog	2	carbohydrate metabolic process
ENSGACT00000027730	0.0222	cox2	MT	copper ion binding; cytochrome-c oxidase activity in mitochondrion & respirasome
ENSGACT00000017921	0.0214	hsp90ab1	18	blood vessel development; leukocyte migration; response to estrogen
ENSGACT00000017116	0.0187	-	8	serine-type endopeptidase activity; proteolysis
ENSGACT00000018389	0.0185	hbe1	11	heme, iron ion, oxygen binding; oxygen carrier activity

## Discussion

Gene co-expression networks play a pivotal role in translating genotype into phenotype. This suggests that phenotypic evolution may often be a consequence of evolution not just of single genes’ protein structure or expression level, but also by changes of co-expression patterns among genes [32,36,37]. For gene co-expression networks to evolve, there must be genetic variations within species that impact the network structure, which selection (or drift) might act on. Therefore, there is a need for methods capable of identifying loci (or chromosomal regions) that are associated with changes in co-expression networks. While methods exist for analysing pairwise gene co-expression [6,8,9,11,42,43], a key challenge lies in methods that can analyze gene co-expression across entire networks.

### Methodological significance

Our snQTL framework offers a methodological advance in network-based association study. Unlike traditional co-QTL methods that test millions of gene pairs independently, snQTL treats the entire co-expression network as a single entity. This dramatically reduces the multiple testing burden. Furthermore, snQTL leverages a tensor spectral statistic that captures the overall signal across the entire network. This approach avoids the need for pre-selecting candidate gene pairs, which can introduce bias. Additionally, unlike regression-based methods that assume an additive genetic effect, snQTL allows for a broad range of genetic effects. The flexibility enables the detection of snQTLs as long as a significant difference exists in co-expression network between genotypes.

The power of snQTL extends beyond co-expression networks. The framework can be generalized to analyze various networks, including microbial networks, proteomic networks, and others. With minor adjustments, snQTL can also handle directed networks like transcription factor binding networks and metabolic network. The core idea of snQTL can be applied for general mapping tasks beyond genetics. For example, the method can handle comparisons of more than three networks, allowing investigation of associations with various discrete factors, such as treatment, location, or environmental conditions.

Several future improvements can be made to snQTL. Currently, snQTL removes all within-chromosome co-expression, since between-chromosome co-expressions are less likely to be generated by LD effects and more likely to indicate functional connections. Future improvements could incorporate recombination maps to identify unlinked markers on the same chromosome and linked markers on different chromosomes, providing a more biologically relevant approach. The other potential extension is on the use of SSTD. The current rank-1 SSTD approximation in snQTL captures the strongest signal in the network difference. Extending this to a higher-rank model could reveal more delicate signals, potentially leading to additional discoveries.

### Biological significance

One of the “grand challenges" of biology is to understand the details of how genotypes produce phenotypes, and thereby develop tools to predict phenotypes. Genotype-phenotype prediction remains a challenge because most phenotypes are the emergent result of complex interactions between numerous genes. Network analyses offer a promising toolkit for representing these complex interactions. Such tools have been applied to gene-gene co-expression data [48–50], single-cell RNAseq data [33], gene-gene epistasis effects [34], proteomic data [35], and beyond, with the goal of describing the logic of genetic regulatory “circuits". The hope is that this network-based approach can reveal rules of life not visible for single genes and their mRNA and protein products, or simple pairwise gene interactions.

Our snQTL analysis of three-spined stickleback gene expression illustrates this potential benefit. We identified three chromosomes with significant snQTLs. Using population genomic data, we were able to identify a candidate gene under especially strong selection within the snQTLs on Chr 18. The gene *ccn6* is a highly pleiotropic gene known to affect growth, metabolism, fibrosis, ROS production, and hence with great potential for network-wide effects in the immune organ sampled for transcriptomics. It appears likely that *ccn6*-mediated changes in electron transport chain function is affecting ROS production differences previously documented between the hybridized populations, with additional consequences for a protective fibrosis phenotype. This gene was not flagged in prior differential expression analyses of the same dataset. Although *ccn6* is expressed at significantly lower levels in Gosling than Roberts Lake fish, the differential expression was not exceptionally large. In contrast, the snQTL (aided by selection scans) makes this gene an important candidate for multivariate phenotypic effects. This result highlights a major limitation in how we currently search for expression-related evolutionary differences: we are most likely to focus on individual loci with large shifts in expression. However, even small changes in expression of one gene can be amplified via downstream effects of entire networks of genes, thereby exerting large phenotypic effects. Scanning large expression networks for correlated changes holds a great promise for uncovering evolving genes whose expression is either highly noisy with respect to genotype, or whose expression is only moderately shifted across populations.

Taken together, our snQTL analysis offers a powerful, effective, and adaptable framework for mapping QTLs that affecting network-based co-expression. We believe our approach brings a broad impact to the genetics community.

## Materials and methods

### Sparse matrix decomposition

We use penalized matrix decomposition [13] to approximately solve for sparse symmetric matrix decomposition in (4). The PDM with input matrix *D* is expressed as


$$\begin{array}{ccc} \underset{∥v{∥}_{2}\leq 1,∥v{∥}_{1}\leq \sqrt{R}}{\max}\text{tr}(D(vv^{T})). & & \end{array}$$ (5)

By [13], the solutions to (5) always have $∥v{∥}_{2}=1$ and satisfy the inequality $∥v{∥}_{1}^{2}\leq ∥v{∥}_{0}\leq R$. Therefore, (5) is an good approximation to sLME in (4). We follow the algorithm in [13] to solve (5).

### Sparse symmetric tensor decomposition algorithm

We solve the optimization problem (2) via SSTD by an iterative algorithm. For a tensor $D\in {\mathbb{R}}^{p_{1}\times p_{2}\times p_{3}}$ and vectors $v^{\left(k\right)}\in {\mathbb{R}}^{p_{k}}$ for *k* = 1 , 2 , 3, we define the tensor-by-vector product on mode 1, mode 2, and mode 3 as


$$\begin{array}{llll} D\times_{1}v^{\left(1\right)} & =\overset{p_{1}}{\underset{i=1}{\sum }}v_{i}^{\left(1\right)}D_{i::},\; & & \; \end{array}$$

$$\begin{array}{llll} D\times_{2}v^{\left(2\right)} & =\overset{p_{2}}{\underset{i=1}{\sum }}v_{i}^{\left(2\right)}D_{:i:},\; & & \; \end{array}$$

$$\begin{array}{llll} D\times_{3}v^{\left(3\right)} & =\overset{p_{3}}{\underset{i=1}{\sum }}v_{i}^{\left(3\right)}D_{::i}.\; & & \; \end{array}$$

Given input tensor *D*, our decomposition algorithm is presented as follows:

1. *Input.* Differential tensor $D\in {\mathbb{R}}^{p\times p\times 3}$, sparsity parameter *R*, and iteration number *T*.
2. *Initialization.* Randomly initilize the unit vectors $v^{\left(0\right)}\in {\mathbb{R}}^{p},u^{\left(0\right)}\in {\mathbb{R}}^{3}$.
3. *For iteration *t* = 1 , … , *T**, alternatively update the decomposition components $v^{\left(t\right)}$ and $u^{\left(t\right)}$:
  $$\begin{array}{ccc} \begin{array}{ccc} v^{\left(t\right)} & =\underset{∥v{∥}_{2}\leq 1,∥v{∥}_{1}\leq \sqrt{R}}{\mathrm{argmin}}\text{tr}(D^{\left(t\right)}(vv^{T})), & \\ \text{ with }D^{\left(t\right)} & =D\times_{3}u^{\left(t-1\right)} \end{array} & & \end{array}$$ (6)
  and
  $$u^{\left(t\right)}=\text{Normalize}(D\times_{1}v^{\left(t\right)}\times_{2}v^{\left(t\right)}).$$
4. *Output.* Output the eigen components $v(D)=v^{\left(T\right)},u(D)=u^{\left(T\right)}$ and estimated sLTE
  $$\Lambda (D)=D\times_{1}v(D)\times_{2}v(D)\times_{3}u(D).$$

Here $\text{Normalize}(v)=v∕∥v{∥}_{2}$ denotes the vector normalization step. We make two comments on our algorithm. Previous work [16] enforces by value truncation. In contrast, our approach achieves sparsity through an optimization process called PMD (Proximal Minimization with Duality) during the update of a variable $v^{\left(t\right)}$ in (6). Our approach is computationally faster and reflects the symmetry in our SSTD model. Second, in our construction of differential tensor input *D*, the third slide $D_{BH}$ can be expressed as the sum of first two slides $D_{AB}$ and $D_{AH}$. While this linear relationship does not affect the final results of association testing, we choose to analyze the model using a full 3-layer tensor *D* for easier interpretation.

### Permutation and empirical p-values

We used permutation to obtain empirical p-values based on our proposed test statistics. Specifically, at each marker, we repetitively shuﬄe three genotypes of samples, re-divide the expression dataset into three groups, and re-calculate the test statistics for *B* times. Let *S* denote the test statistic with original genotype, and $S_{b}$ denote the test statistic with shuﬄed dataset in the *b*-th permutation for *b* = 1 , … , *B*. We obtain the empirical p-value as


$$\text{p-value}=\frac{1}{B}\overset{B}{\underset{b=1}{\sum }}I\{S_{b}\geq S\},$$

where *I* { ⋅ }  is the indicator function.

In our stickleback data analysis, we first obtained the empirical p-values for all markers with $B_{0}=100$ permutations for preliminary snQTL screening. For the markers showing preliminary empirical p-values smaller than 0.05, we re-ran the tests with *B* = 500 permutations for accurate p-values estimations.

### Sparsity hyperparameter

We discuss the tuning of the sparsity hyperparameter *R*. A higher *R* leads to denser connection in the estimated joint differential network. Common methods for selecting *R* include cross-validation via random test-train splits. Other works [13,19] suggested setting *R* proportional to the feature dimension *p*. We recommend exploring a range of *R* and choose the one that balances the number of discoveries with their biological relevance.

For simulations in Fig 2C, we use a non-sparse setting (*R* = *p*) for a fair comparison with the non-sparse local method. Additional experiments with varying sparsity parameter are detailed in *S1 Appendix*. Our snQTL method shows a stable performance across a wide range of *R*. For the stickleback data analysis, we set the sparsity parameter *R* to 0.09*p*. This aligns with the expectation that only a few thousand genes contributing to the main co-expression differences. In other applications, we recommend choosing *R* based on background knowledge and the scientific context.

In addition, we propose a data-driven approach for selecting *R* when no prior information is available. While our snQTL objective function (2) is not built on probabilistic distributional assumptions, following earlier works in similar contexts [2,16], we suggest using a Bayesian Information Criterion (BIC)-based approach for parameter tuning. Given the differential tensor $D\in {\mathbb{R}}^{p\times p\times 3}$, we choose the sparsity parameter *R* that minimizes the following BIC:


Rselect=argmin1≤R≤p3p2 log ⁡  (∥D−ΛRvR∘vR∘uR∥F23p2 )+(2+2∥vR∥0)log ⁡ 3p2, (7)

where $\left(\Lambda^{R},v^{R},u^{R}\right)$ are the SSTD components with the sparsity parameter *R*. The first term in (7) represents the squared loss of the SSTD decomposition, and the second terms is the penalty proportional to the number of parameters.

This BIC metric (7) balances the approximation error and sparsity, and it has been widely used for hyperparameter selection in tensor literature [1,3–5,16]. We performed simulations to show the efficacy of the proposed approach; see *S1 Appendix* for details. We also applied the BIC selection to the real stickleback data. While the selected sparsity parameters *R* for some markers exceed 0.09*p*, all testing results and joint differential network estimation conclusions remain the same across different choices of *R*. This consistency indirectly verifies the robustness of our current stickleback analyses. See *S1 Appendix* for additional simulation results and stickleback data analysis.

## Supporting information

S1 Appendix The S1 Appendix contains extra analyses and details for simulation and stickleback data. For the simulated data, the appendix includes synthetic data generation, correlation map discussions, and the simulation results for more generations. For the stickleback data, the appendix includes the pre-processing steps, test results with matrix statistics, snQTL analyses results on Chr 3 and Chr 8, and test results that account for tape worm infection.
(PDF)
