# Supplementary material for: A spectral framework to map QTLs affecting joint differential networks of gene co-expression
Source: PLoS Comput Biol. 2025 Apr 17;21(4):e1012953. doi: 10.1371/journal.pcbi.1012953 (PMC12040279; doi:10.1371/journal.pcbi.1012953)
Supplement: S1 Appendix — For the simulated data, the appendix includes synthetic data generation, correlation map discussions, and the simulation results for more generations. For the stickleback data, the appendix includes the pre-processing steps, test results with matrix statistics, snQTL analyses results on Chr 3 and Chr 8, and test results that account for tape worm infection. (PDF) [file pcbi.1012953.s001.pdf]

S1 Appendix for A spectral framework to map QTLs affecting joint differential networks of gene co-expression

Jiaxin Hu[1], Jesse N. Weber[2], Lauren E. Fuess[3], Natalie C. Steinel[4], Daniel I. Bolnick[5], and Miaoyan Wang[1]

[1]Department of Statistics, University of Wisconsin-Madison

[2]Department of Integrative Biology, University of Wisconsin-Madison

[3]Department of Biology, Texas State University

[4]Department of Biological Sciences, University of Massachusetts Lowell

[5]Department of Ecology and Evolutionary Biology, University of Connecticut

A Extra analyses of simulated data

A.1 Simulated data generation

Our simulated dataset consists of both genotypes and expressions of p genes for n samples, represented as two n-by-p matrices G and E, respectively. To simulate genotypes, we started with homozygous parents (one with genotype AA and the other one with BB) and simulated the genotypes for an F1 cross with n samples followed by an F2 intercross generation with random chromosomal crossing overs. All F1 samples have heterozygous AB genotypes for all genes. We randomly select two individuals from the n F1 samples as parents, generate two gametes from these parents, and combine the gametes to get the genotype for one F2 individual. We repeat this step n times to get n F2 samples. For each F1 gamete, we simulate one recombination event per chromosome with a probability 0.7, randomly placed along the chromosome with a uniform distribution. Due to the homozygous parents, the minor allele frequency in this study is 0.5. For extra analysis in Section A.5, we repeated the breeding procedures to simulate genotypes for F3, F4, and F5 intercross generations, to mimic the process of generating fine-mapping populations. The simulated genotype matrices have entries with values 0, 1, or 2, where 0 and 2 refer to homozygous genotypes, and 1 refers to the heterozygous genotype. We use i for the index of gene and use j for the index of sample.

Given the simulated genotype matrix G, we simulate baseline expression level (null data) for each gene in each sample, assuming no network effects. Specifically, we simulated the null expression value of the i-th gene for the j-th sample independently based on Poisson

distribution with scalar parameters $(\mu, \sigma_\alpha, \sigma_\beta)$:

$$E^0_{ji} \sim_{\text{ind}} \text{Poi}(\exp(\alpha_i + \beta_j \times G_{ji})), \quad \text{where } \alpha_i \sim_{i.i.d.} N(\mu, \sigma^2_\alpha),\ \beta_i \sim_{i.i.d.} N(0, \sigma^2_\beta).$$

The parameters μ and σ^2_α control the overall mean and deviation of the expression data, respectively, and σ^2_β controls the degree of genetic effects on expression. Next, we constructed the gene co-expression networks, represented as symmetric matrices $M_k \in \mathbb{R}^{p \times p}, k = 0, 1, 2$, for three genotypes. For genotype k, the entries in M_k follow the following distribution independently with scalar parameters (d_k, δ_k):

$$M_{k,i_1,i_2} = \text{Bernoulli}(d_k) \times N(0, \delta^2_k), \quad \text{where } 1 \leq i_1 \leq i_2 \leq p, \text{ and } M_{k,i_1,i_2} = M_{k,i_2,i_1}.$$

The parameter d_k controls the sparsity of gene-gene correlation (i.e. number of nonzero entries in M_k), and δ^2_k controls the magnitude of nonzero entries in M_k in genotype k. The genetic-related network effects are specified by setting various values of (d_k, δ^2_k). Furthermore, to mimic the additive genetic effects, we may set the heterozygous network as the average of homozygous networks, i.e., $M_1 = (M_0 + M_2)/2$. For simplicity we do not consider more complex patterns of dominance or over-/under-dominance, which may exsit in empirical co-expression networks in hybrid populations.

Last, we imposed the co-expression network effect on top of the the null expression. We randomly selected one gene, say i^*, as the snQTL. The genotype of snQTL i^* determined which matrix M_k is for the co-expression network effect. We alter the the expressions for genes involved in co-expressed pairs, using the following calculations:

$$E_{ji} = E^0_{ji} + \sum_{l \leq i} M_{G_{ji^*},li} \times (E^0_{ji} - \exp(\alpha_l + \beta_l \times G_{jl})),$$

where the second term on the right-hand side is the network effect of snQTL i^* to the expression of gene i in sample j. It is possible for above equation to generate negative expressions, denoted as E^N_{ji}, which is impossible in real life. The simplest way is to set negative expressions as 0; however this simple way leads the undesired concentration around 0 compared with real data. Therefore, we consider the re-generation for negative expression:

$$E_{ji} \sim_{\text{ind}} \text{Poi}(\exp(\mu + \eta * E^N_{ji})),$$

where η is a positive constant. Intuitively, if we have a negative expression with larger absolute value, we will have a low expectation in above Poisson distribution.

In our simulation, we generated data of $p = 200$ genes located on 20 chromosomes with varying population size n from 50 to 500. We considered additive network effect and tuned the parameters to mimic the distribution of expression levels from the empirical stickleback dataset, by choosing:

$$\mu = 2.6,\ \sigma_\alpha = 1,\ \sigma_\beta = 0.75,\ d_0 = 0.01,\ \delta_0 = 0.5,\ d_2 = 0.03, \delta_2 = 5,\ \eta = 0.05.$$

A.2 Raw correlation map for simulated data

Our analysis compared the absolute genetic correlation heatmaps between real stickleback data [**?**] and synthetic data (Figure S1). Unlike Figure 2A (real data), the synthetic heatmap shows correlations ranging from -1 to 1, while real marker pairs mostly have positive correlations. This difference arises from three aspects of our simulation design:

- Parent Genotype Initialization: In the simulation, half of first-generation diploid parents are given genotype AA while the other half are given genotype BB, for all markers. This leads to all offspring in the first generation having the same genotype AB for all markers.

- Enforced Crossover: During gamete formation, we simulate chromosomal crossover events. While these crossovers are important for real breeding, they can create negative correlations in our simulated data. This is because markers near the head and tail of the chromosome are likely to have different genotypes in the resulting offspring, even though they came from the same parent.

- Limited Breeding Generations: We only simulated breeding for two generations (F1 and F2). With more generations, these negative correlations due to initial crossovers would eventually be diluted through further recombination.

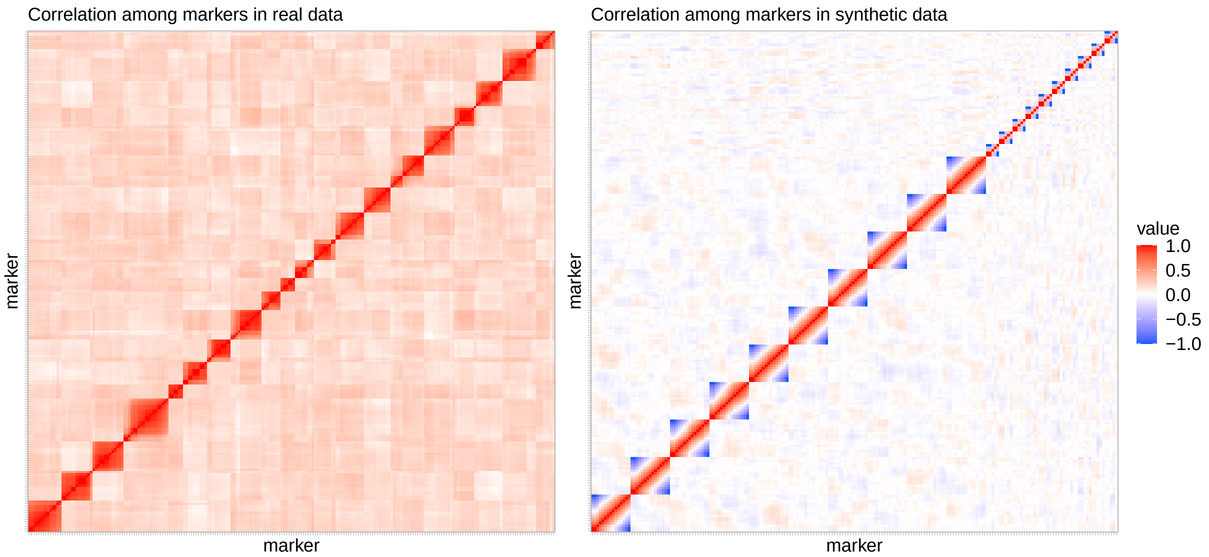

Figure S1: Genetic correlation heatmap among markers in real F2 hybrid three-spined stickleback data and synthetic data. Blue color indicates negative correlation; red color indicates positive correlation.

For example, consider a chromosome with 5 markers, with 0 representing genotype A and 1 representing genotype B. The second-generation diploid parents have two chromatids for chromosome 1, with genotype (00000) and (11111). With a crossover at the fourth marker, possible gametes could be (00011) and (11100). Combine two random gametes to obtain the genotype of diploid offspring. If marker 1 has genotype (0,0), then marker 5 must have (1,1); if marker 1 has genotype (0,1), then marker 5 must have (1,0). Markers at opposite ends of the chromosome have opposite genotypes, leading to a negative correlation.

It is important to note that our snQTL testing is invariant to the label switch between A and B. Therefore, the presence of negative correlations in the simulated data does not

impact the evaluation of our method's effectiveness.

A.3 Detailed simulation results for Fig 2C

Table S1 shows the average p-values for all methods in simulation Figure 2C. As the population size increases, p-values for all methods decrease. However, the improvement in accuracy for the local approach is minimal compared to the rapid increase in accuracy observed with snQTL.

Method / Population Size	50	80	100	150	200	250	300	500
Max at snQTL	0.397	0.272	0.221	0.154	0.131	0.113	0.080	0.008
Tensor at snQTL	0.399	0.291	0.230	0.154	0.133	0.112	0.080	0.009
Local at snQTL	0.746	0.723	0.700	0.675	0.621	0.683	0.641	0.597
Max at non-snQTL	0.517	0.493	0.518	0.457	0.472	0.438	0.494	0.444
Tensor at non-snQTL	0.523	0.491	0.515	0.459	0.474	0.430	0.497	0.453
Local at non-snQTL	0.822	0.842	0.862	0.869	0.860	0.913	0.909	0.923

Table S1: Averaged p-values in simulation Figure 2C.

A.4 Simulation with varying sparsity parameters

Figure S2 presents additional simulation results with snQTL using different sparsity parameter. Following earlier works [? ?], we set $R = c^2 p$, where $c \in [0, 1]$ is the tunning parameter. Our snQTL shows a stable performance regardless the choice of sparsity. The accuracy of snQTL keeps over 0.7 for all sparsity levels. The only exception is the extremely sparse case with $c = 0.1$ letting only 1% genes contribute to the joint different network. However, the synthetic data has the true sparsity level around $c^2 = \sqrt{d_2} = \sqrt{0.03} = 17\%$ ($c = 0.42$), where d_2 is the network density in Section A.1. Combining with the main simulation in Fig 2, we conclude that our method provides a substantial and robust increase in performance with and without sparsity constraints, compared with the local method.

To test the efficacy of the data-drive BIC approach, we further consider the simulation with synthetic differential tensors $\mathcal{D}$ of different sparsity levels. Specifically, with $p = 500$,

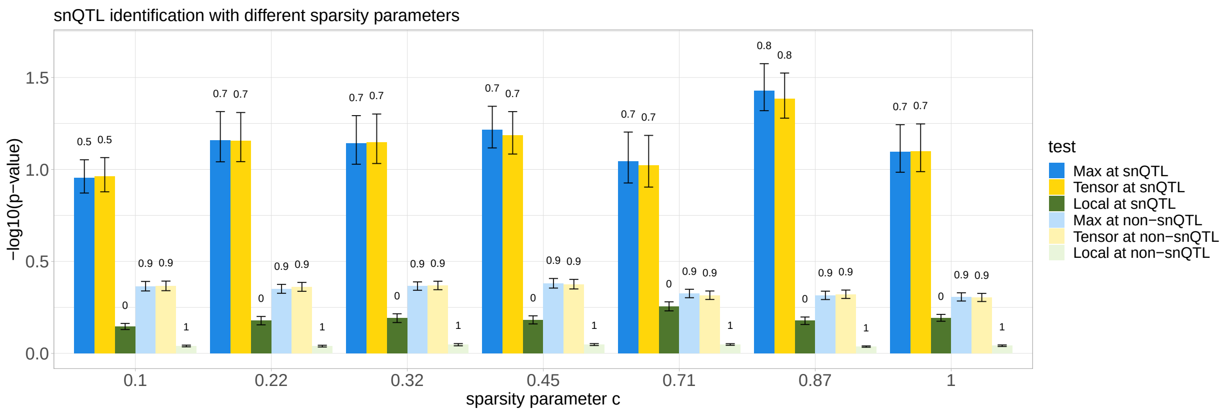

Figure S2: Barplots comparing the snQTL identification performances for snQTL framework and local method (F-test for regression of pairwise co-expression onto genotype) on synthetic data with varying sparsity parameter $c \in [0.1, 1]$. The population size is set to 300. A larger c results in more genes contribute to the co-expression changes. True positive (or negative) rates for the tests at snQTL (or non-snQTL) are shown above the bars. All reported numbers are averaged across 50 replications for each population size.

we generate $\mathcal{D} \in \mathbb{R}^{p \times p \times 3}$ as

$$\mathcal{D} = \Lambda v^c \circ v^c \circ u + \mathcal{E},$$

where $\Lambda = 25$, v^c, u have normalized entries from Uniform$(-1, 1)$ such that $\|v^c\|_0 = c^2 p$, $\|v^c\|_2 = \|u\|_2 = 1$, and $\mathcal{E} \in \mathbb{R}^{p \times p \times 3}$ is the noise tensor with 3 symmetric p-by-p slides consisting normal distribution entries from $N(0, 0.01)$. The rough signal-to-noise ratio $\|\Lambda v^c \circ v^c \circ u\|_F / \|\mathcal{E}\|_F$ is smaller than 0.1, which mimics the small signal cases in practice. We vary the sparsity level with $c^2 = 0.05, 0.1, \ldots, 0.7$ and consider the candidates from 0.01 to 0.8. Table S2 indicates the efficacy of our selection approach, especially for the sparse case with $c^2 \leq 0.5$. Note that in SSTD algorithm, our algorithm optimizes the objective under a relaxed convex restriction, $\|v^c\|_1 \leq c\sqrt{p}$. Thus, for SSTD outputs, c^2 is a lower bound for the proportion non-zero elements in v^c. This observation explains the BIC selection results under the relatively dense cases $c^2 > 0.5$. Though we select c's smaller than the ground truth, the actual number of non-zero elements in v^c is larger than $c^2 p$ and well-approximate the true signal. Hence, we conclude that the proposed BIC approach is an effective for sparsity selection in principle.

A.5 Simulation for synthetic data with more generations

Figure S3 presents additional simulation results using synthetic data generated from F3, F4, and F5 hybrid crosses. These later breeding generations are often used in fine-mapping

True c^2	0.05	0.1	0.2	0.3	0.4	0.5	0.6	0.7
Selected $\hat{c}^2$	0.05 (0)	0.1 (0)	0.2 (0)	0.3 (0)	0.4 (0)	0.5 (0)	0.51 (0.02)	0.6

Table S2: Sparsity parameter selection with BIC approach. We vary the proportion $R = c^2 p$ with $c^2 = 0.05, \ldots, 0.7$. BIC approach considers candidate c^2 from 0.01 to 0.8. For each true c^2, we repeat the simulation for 20 times and report the average and standard deviation of the selected c^2.

studies because they create smaller linkage windows. In essence, these later generations move closer to mimicking GWAS in genetically diverse outbred populations, where breeding is not involved. We find that our comparison conclusions remain the same to those performed with the F2 hybrid data in the main text. This consistency demonstrates that snQTL maintains its outer-performance compared to the local method, regardless of the breeding generation used in the simulation.

A.6 Simulation for GWAS-like synthetic data

We also generate GWAS-like synthetic data to show the general efficacy of snQTL. Genotype generation is the main difference between F2 and GWAS-like data. For GWAS-like synthetic data, we generate genotype with random minor allele frequency and Hardy-Weinberg distribution. Specifically, we generate the genotype for the i-th gene of the j-th sample as

$$G_{ji} \sim \text{Hardy-Weinberg}(\gamma_i), \text{ with } \gamma_i \sim \text{Uniform}[0.1, 0.9],$$

independently for $i = 1, \ldots, p, j = 1, \ldots, n$. Here, for $X \sim \text{Hardy-Weinberg}(\gamma)$, we have

$$\mathbb{P}(X = 0) = (1-\gamma)^2, \quad \mathbb{P}(X = 1) = \gamma(1-\gamma), \quad \mathbb{P}(X = 2) = \gamma^2.$$

Given GWAS-like genotype, we generate the expression following the same procedure used in F2 hybrid in Section A.1.

Figure S4 indicates that GWAS-like genotype does not show LD structure as real data, while the expression distribution of GWAS-like data in Figure S5 is close to real data. The simulation comparsion results in Figure S6 still supports the superior performance of our snQTL compared with the local method. Meanwhile, snQTL shows a less power than that for F2 hybrid (Figure 2). The decrease in power is expected, since GWAS genotype generation will lead more unbalanced genotype group partitions with small or large γ.

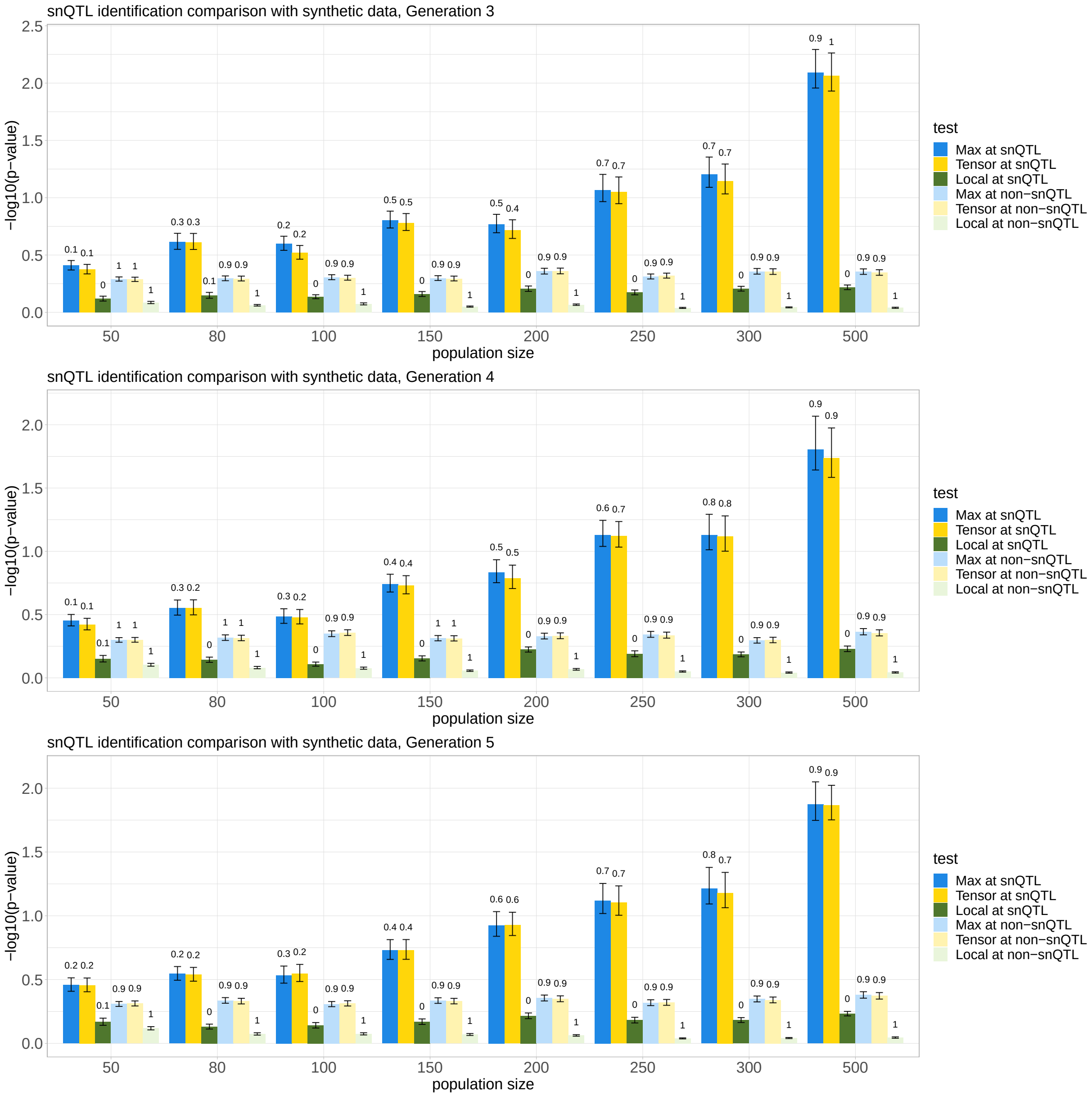

Figure S3: Comparison between snQTL framework and local methods (F-test for regression of pairwise co-expression against genotype) on synthetic data with varying population size from 50 to 500. Synthetic datasets are generated from the F3, F4, and F5 hybrids (from top to bottom), respectively. True positive (or negative) rates for the tests at snQTL (or non-snQTL) are shown above the bars. All reported numbers are averaged across 50 replications for each population size.

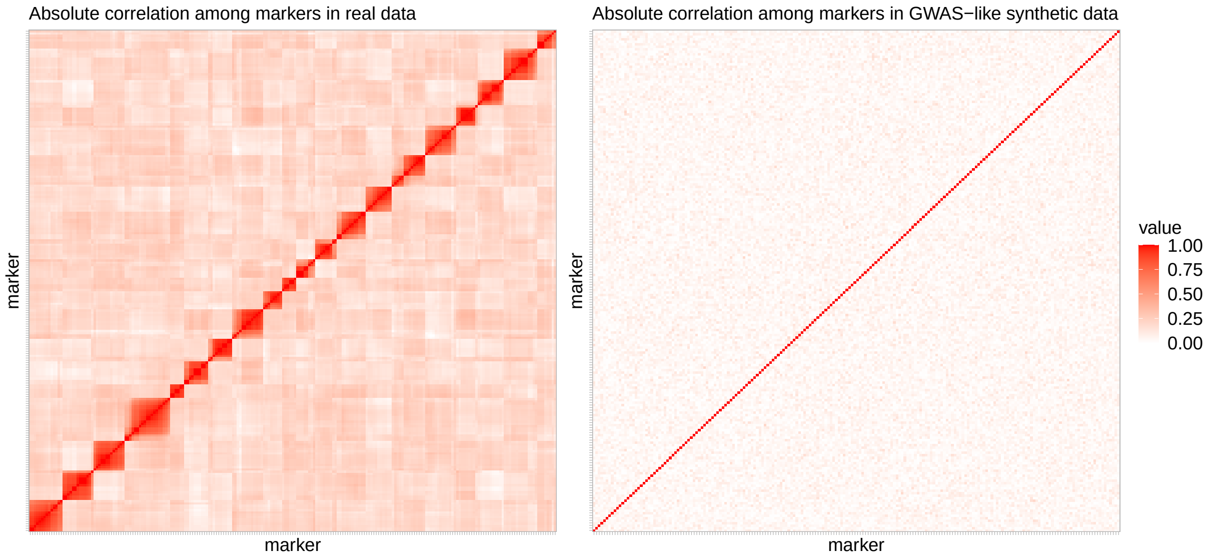

Figure S4: Absolute genetic correlation heatmaps among the markers in real F2 hybrid three-spined stickleback data [?] and GWAS-like synthetic data. Markers are ordered following their positions on the genome. Genetic correlations are measured by absolute sample Pearson correlation coefficients between the genotypes of two markers.

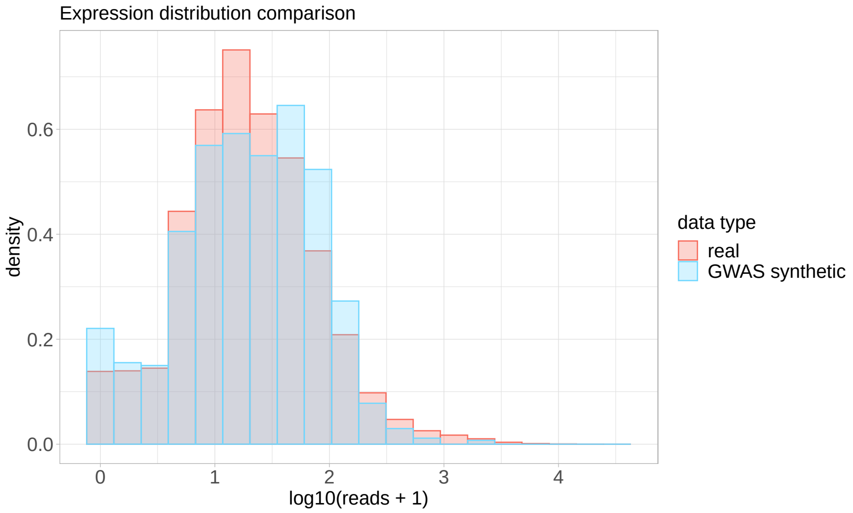

Figure S5: Density histograms for expression counts in real stickleback and GWAS-like synthetic data. The parameters in GWAS-like synthetic data generation are the same for Fig 2 in the main text.

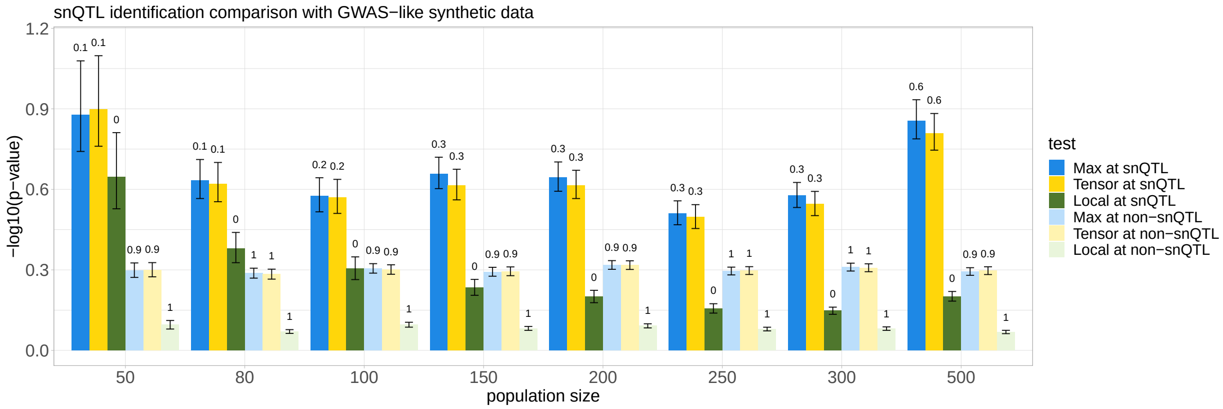

Figure S6: Barplots comparing the snQTL identification performances for snQTL framework and local method (F-test for regression of pairwise co-expression onto genotype) on GWAS-like synthetic data with varying population size from 50 to 500. We set sparsity parameter $R = p$ in snQTL for a fair comparison with non-sparse local method. True positive (or negative) rates for the tests at snQTL (or non-snQTL) are shown above the bars. All reported numbers are averaged across 50 replications for each population size.

B Pre-processing and extra analyses with stickleback data

B.1 Pre-processing of stickleback data

The original stickleback data [?] includes gene expression levels (transcript counts) for 26,285 genes and genotypes for 234 genetic markers from 351 samples in the F2 and backcross generations. The data also contains covariate information such as sex, family, and infection status.

We describe our pre-processing on the gene expression matrix. Let X^0 denote the raw transcript count matrix, and $n = 351, p = 26,285, q = 234$ for the numbers of samples, genes, and markers, respectively We use i for the index of gene and use j for the index of fish.

We follow these three main steps:

(i) Normalization. We normalized the raw transcript counts to account for differences in sequencing depth across samples. This is achieved by dividing each transcript count X^0_{ji} by the total transcript count for that sample. The resulting normalized matrix is denoted by X^N with entries

$$X^N_{ji} = \frac{X^0_{ji}}{\sum_{i=1}^{p} X^0_{ji}}, \quad \text{for sample } j = 1, \ldots, n.$$

The denominator $\sum_{i=1}^{p} X_{ji}^0$ is referred to as the library size or sequencing depth of the sample j.

(ii) Covariate Removal. We removed the effects of covariates of sex and family. This was done using linear regression. The covariate sex has three levels (female, male, and non-identified), and the covariate family has 36 levels based on the parental origin. The residuals from this model represent the gene expression levels after removing the influence of these covariates.

$$X_{ji}^R = \text{residual}(X_{ji}^N \sim S_j + A_j), \quad \text{for gene } i = 1, \ldots, p.$$

Here, $y \sim x$ represents the standard repression model in which y servers as the response and x serves as predictor, and S_j denotes the sex effect and A_j denotes the family effect. We use X^R to denote the residual matrix after removing covariate effects.

As we mentioned in the main text, we also consider warm infection status as a possible covariate. We explored this further in Section B.3 using a similar regression approach with an additional binary predictor for worm presence/absence:

$$X_{ji}^R = \text{residual}(X_{ji}^N \sim S_j + A_j + W_j), \quad \text{for gene } i = 1, \ldots, p,$$

where W_j is a binary predictor encoding the worm presence/absence of the samples.

(iii) Gene Selection: Finally, we selected the top 10,000 genes with the highest adjusted mean expression m_i defined by

$$m_i = \frac{1}{n} \sum_{j=1}^{m} X_{ji}^R, \quad \text{for all genes } i = 1, \ldots, p$$

This metric represents the average expression level for each gene across all samples. We focused on highly expressed genes because they are more likely to be relevant for biological processes. The cutoff of 10,000 genes was chosen for computational efficiency.

B.2 Testing results with matrix statistics

In addition to the tensor statistics, we investigated two alternative approaches based on matrix-spectrum statistics. The first one uses the max statistic defined in the main text. The second approach utilizes a variant called the sum statistic, defined as:

$$\text{Stat}_{\text{sum}} = \lambda(D_{AB}) + \lambda(D_{AH}) + \lambda(D_{BH}),$$

where $\lambda(\cdot)$ represents the sLME, D_{AB}, D_{AH}, and D_{BH} represent pairwise differential network from the original sample correlation matrices.

As shown in Figures S7 and S8, we found that both the max and sum statistics identified snQTLs for sticklebacks clustered on chromosomes 3, 8, and 18. This consistency across different statistical methods strengthens the reliability of our findings.

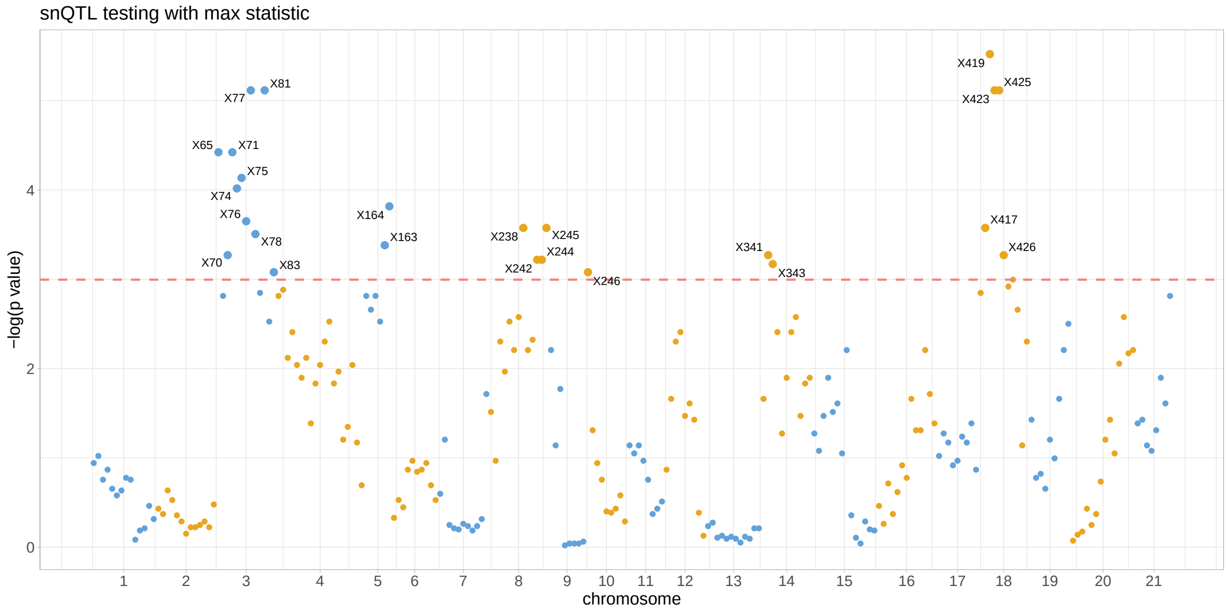

Figure S7: Manhattan plot for snQTL testing with max statistics marks stickleback snQTLs (above pink dashed line with p-values smaller than 0.05), mainly clustered in Chr3, Chr8, and Chr18.

B.3 Testing results controlling for tape worm infection

To prioritize analyses with limited computational resources, we performed snQTL testing only on the top snQTLs identified from the non-infection-controlled expression data. We found that the top snQTLs on chromosomes 3, 8, and 18 remained the same even after controlling for infection status (Table S3). This consistency suggests that the network effects

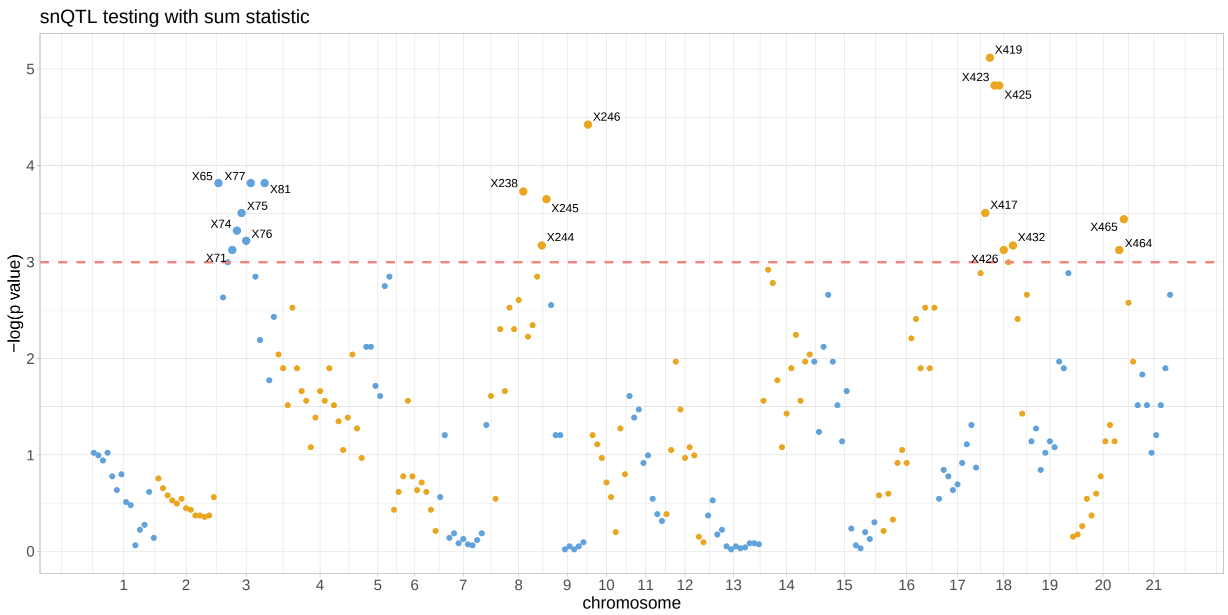

Figure S8: Manhattan plot for snQTL testing with sum statistics marks stickleback snQTLs (above pink dashed line with p-values smaller than 0.05), mainly clustered in Chr3, Chr8, and Chr18.

of these snQTLs are not driven by the environmental factor of worm infection. Rerunning the analysis with infection-controlled expression data for all markers might reveal even more significant snQTLs and related discoveries. However, we will leave such additional analyses for future studies.

Markers	p-values (infection uncontrolled)	p-values (infection controlled)
X77	0.01	0.002
X81	0.012	0.004
X419	0.008	0.004
X423	0.008	0.008
X238	0.018	0.024
X245	0.02	0.016

Table S3: Empirical p-values of top snQTLs obtained by snQTL testing with infection-uncontrolled expression (main text result) and with the infection-controlled expression (Section B.3).

B.4 Testing results excluding cross type effects

In current stickleback data pre-processing, we exclude the family effects by regressing the normalized expression onto the family covariate with 36 levels (Section B.1). In real data,

the cross type information (with 3 levels, GBC, RBC, F2) for each sample is also recorded, and family covariates are nested in cross type covariates. In principle, the analysis results excluding family effects are more conservative than that excluding cross type effects. Hence, we also investigate the snQTL results only without cross type effects. Table B.4 indicates that top snQTLs on chromosomes 3, 8 remain significant while snQTLs on chromosome 18 is perturbed by different pre-processing procedures. For the most notable identified snQTL, X419, the corresponding gene leverage (Figure S9) and joint differential network analysis without cross type effects show similar patterns as that without family effects (presented in the main text). Therefore, in this paper, we stick with the conservative family-covariate pre-processing. We leave the additional full analysis with cross-type-covariate pre-processing in the future.

Markers	p-values (without family effects)	p-values (without cross type effects)
X77	0.01	0.02
X81	0.012	0.022
X419	0.008	0.052
X423	0.008	0.044
X238	0.018	0.138
X245	0.02	0.182

Table S4: Empirical p-values of top snQTLs obtained by snQTL testing without family effects (main text result) and without cross type effects (Section B.4).

B.5 Population branch statistic distributions on Chr 3 and Chr 8

Figures S10 and S11 show that several protein-coding genes lie in regions adjacent to PBS outliers near the snQTLs on Chr 3 and Chr 8.

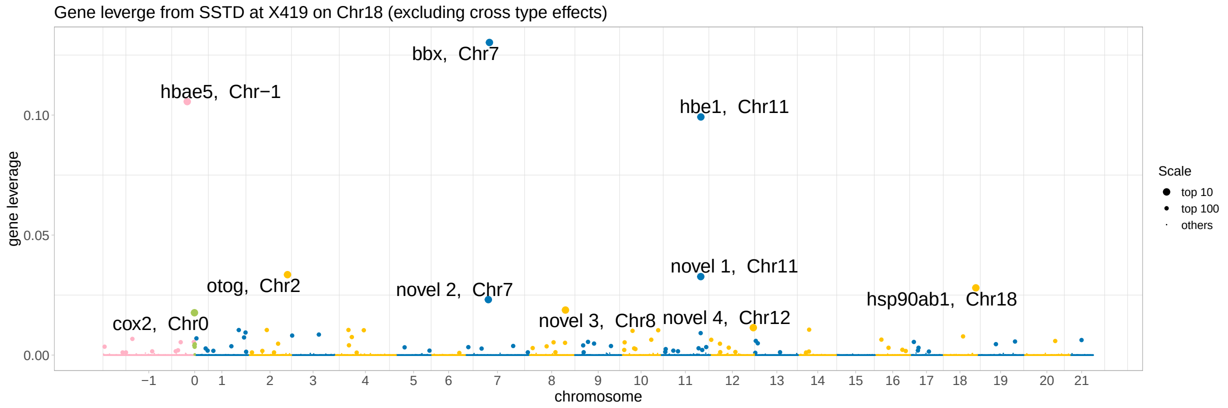

Figure S9: Joint differential network analysis at snQTLs X419 on Chr 18 with leverage scores for 10000 genes, excluding the cross type effects. Primary genes with top 10 leverage are highlighted with transcription IDs. Mitochondrial genome (MT) and scaffold region are coded as Chr 0 and Chr -1, respectively. novel 1: ENSGACT00000018413; novel 2: ENSGACT00000026589; novel 3: ENSGACT00000017116; novel 4: ENSGACT00000017388.

B.6 Joint differential networks for snQTLs on Chr 3 and Chr 8

We further analyzed the top snQTLs identified earlier: X77 on Chr 3 and X238 on Chr 8. Interestingly, the results at these loci were highly similar to those observed at X419 on Chr 18 (see main text). Figure S12 illustrates this similarity. The sets of top 100 genes identified by high tensor leverages at X77 and X238 show a significant overlap with the top genes for X419, especially the top 10 most important genes. However, the specific ranking of these genes might differ slightly between snQTLs. To explore this further, we constructed a joint differential network for X77 and X238, using the top genes identified at X419. Figure S13 reveals the strong connections between primary and secondary genes. This patten is consistent across these multiple snQTLs. This consistency strengthens the evidence that our findings regarding oxygen transport pathways in the joint differential network are not simply random observations.

B.7 eQTL analysis at snQTL X419

We perform the eQTL analysis at our most significant snQTL, X419 on Chr 18. We regress the residual expression for the top 10,000 genes (used for snQTL main text analysis) onto the genotype of X419. The eQTL results are shown in the Manhattan plot Figure S14.

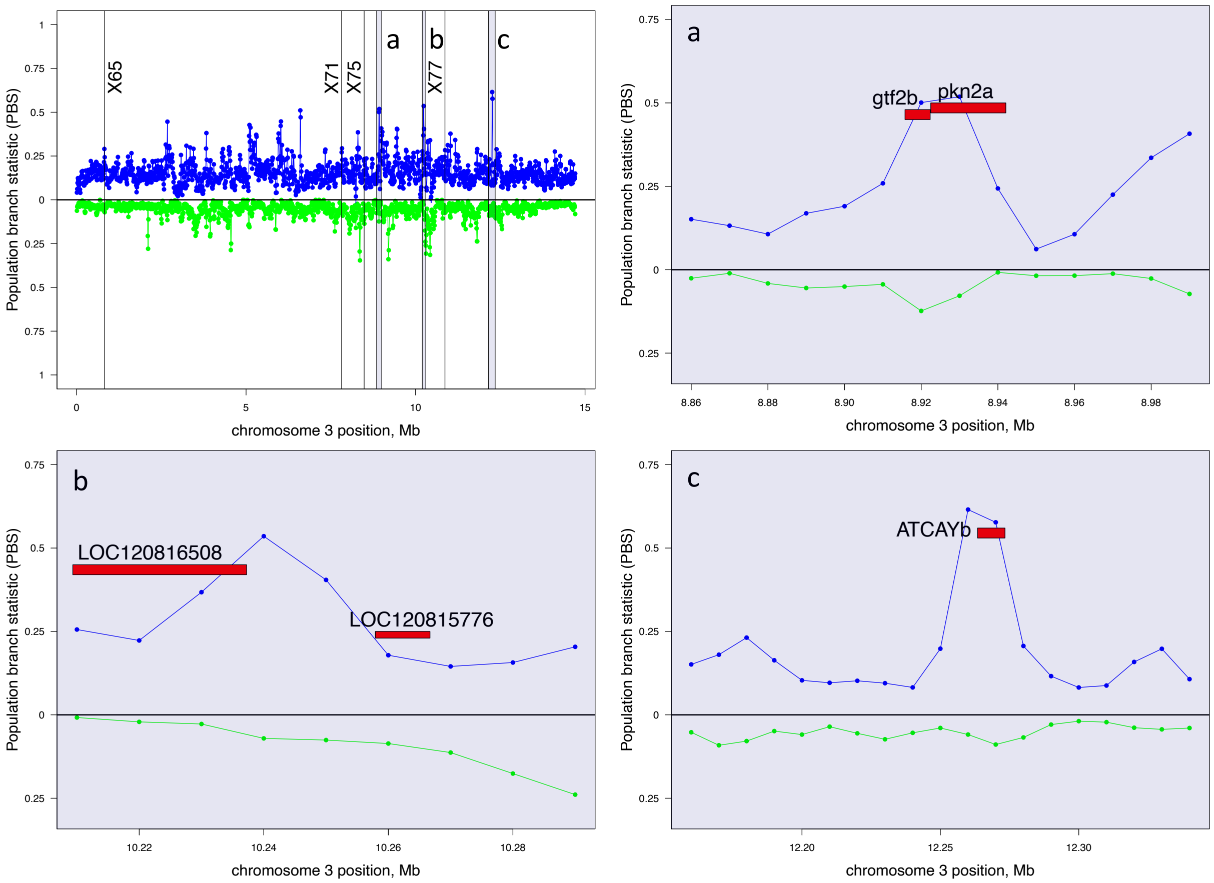

Figure S10: Strong genomic targets of selection with high population branch statistic distribute around the outstanding snQTLs in Chr 3. Values above the medial line represent higher PBS in Gosling Lake (blue); values below the line represent higher PBS in Roberts Lake (green). Protein-coding genes lie in regions adjacent to three PBS outliers (a, b, c) around snQTLs (markers X65, X71, X75, and X77).

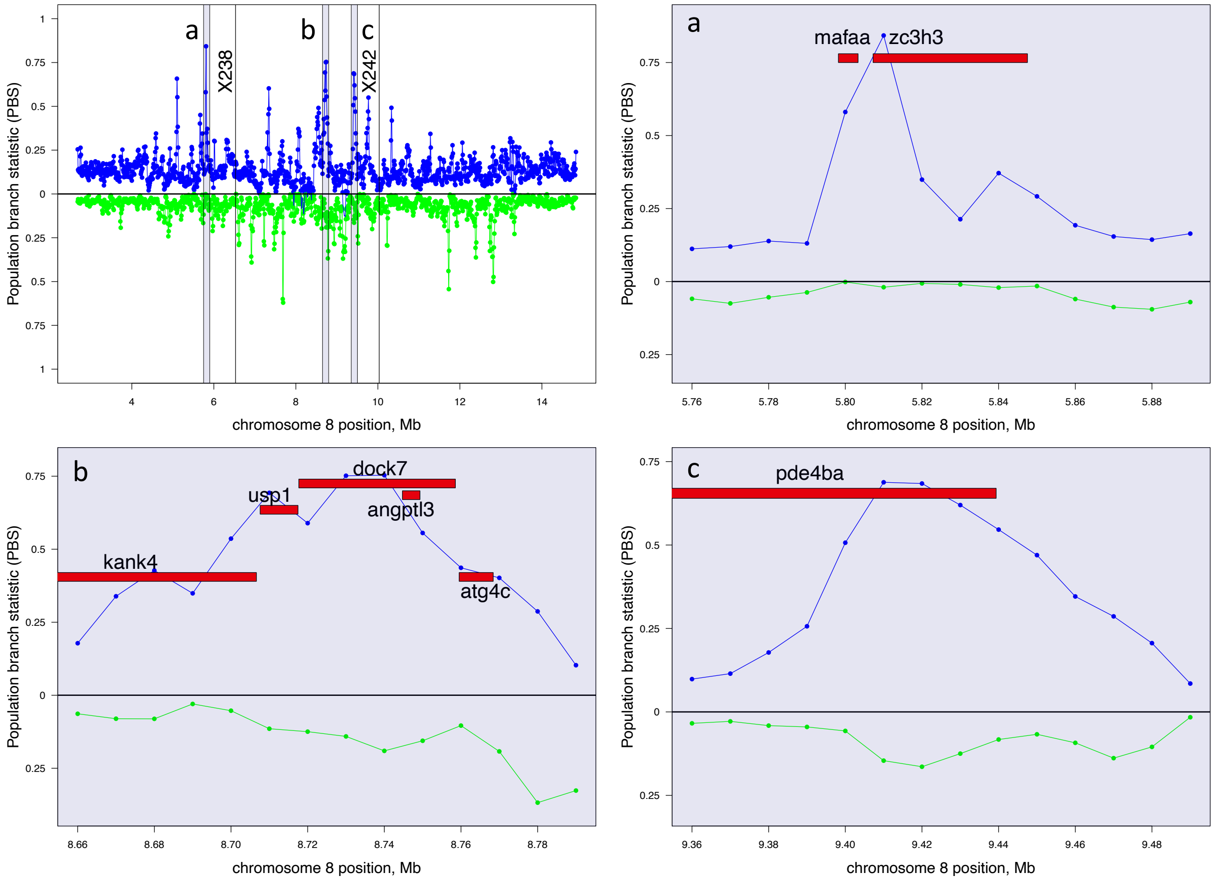

Figure S11: Strong genomic targets of selection with high population branch statistic distribute around the outstanding snQTLs in Chr 8. Values above the medial line represent higher PBS in Gosling Lake (blue); values below the line represent higher PBS in Roberts Lake (green). Protein-coding genes lie in regions adjacent to three PBS outliers (a, b, c) around snQTLs (markers X238 and X242).

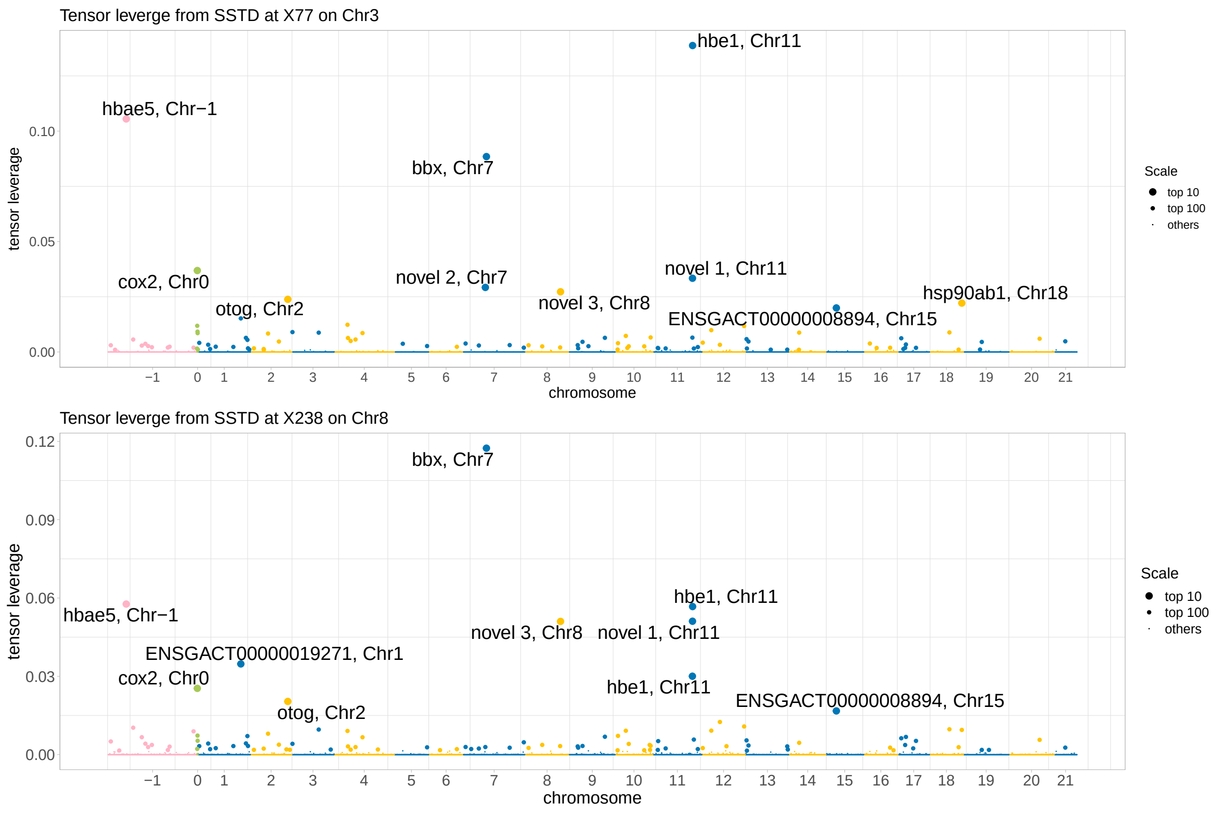

Figure S12: Joint differential network analysis at snQTLs X77 on Chr 3 (top) and X238 on Chr 8 (bottom) with leverage scores for 10000 genes. Primary genes with top 10 leverage are highlighted with transcription IDs. Mitochondrial genome (MT) and scaffold region are coded as Chr 0 and Chr -1, respectively.

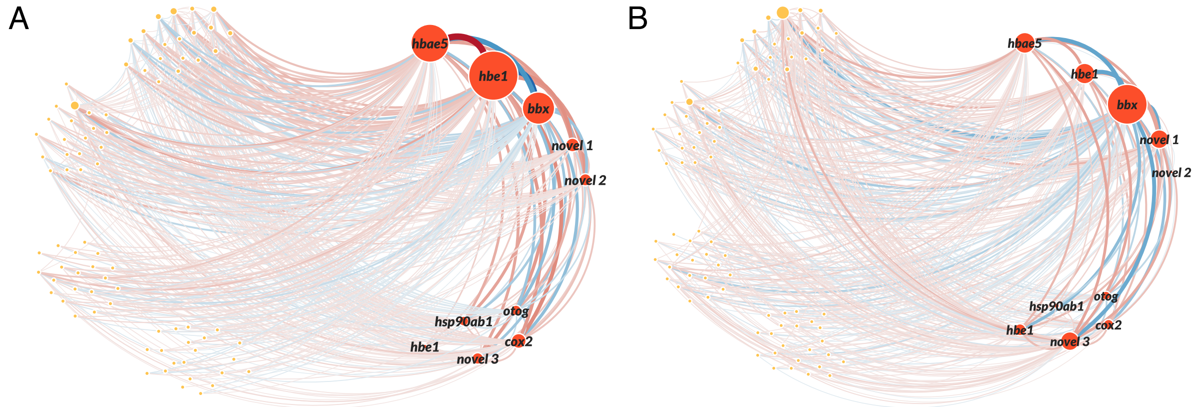

Figure S13: Joint differential networks at snQTLs (A) X77 on Chr 3 and (B) X238 on Chr 8 with top 100 genes identified in the analysis at X419 on Chr 18. The edge width indicates the connection strength between two genes; the diameter of node indicates the leverage of the genes; the color indicates the enhancement (red) or reduction (blue) of the connection compared with average level. Top 10% strongly connected edges are selected. novel 1: ENSGACT00000018413; novel 2: ENSGACT00000026589; novel 3: ENSGACT00000017116.

After multiple testing Bonferroni correction with critical value $0.05/10,000$, only 10 genes (marked on the plot) have expressions significantly affected by X419 genotype. We can conclude that X419 is a cis-eQTL but not a tran-eQTL.

Further, we test for and found a marginally significant ($p = 0.051$) association between *ccn6* expression and X419 genotype within the cross type. Figure S15 indicates the genotype effects to the averaged *ccn6* expression levels.

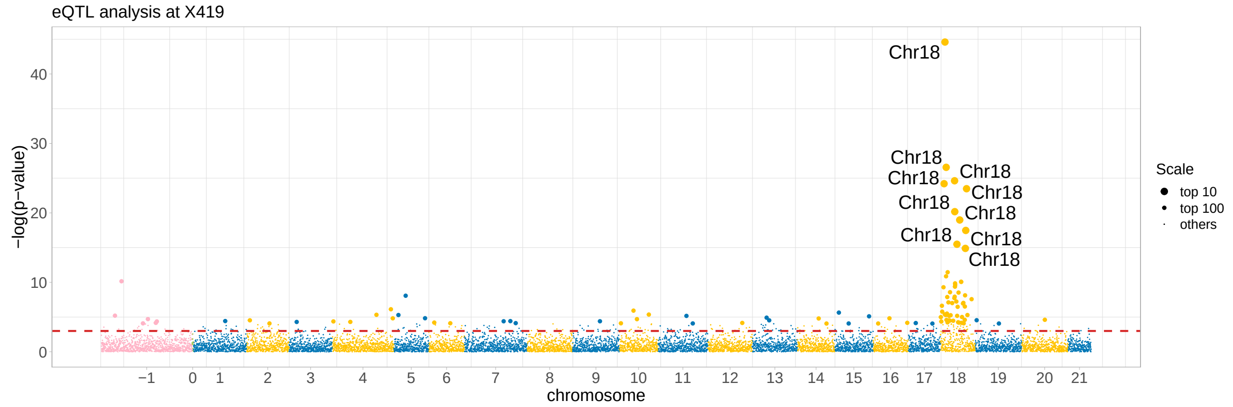

Figure S14: eQTL analysis at snQTL X419. The red dashed line refers to the critical threshold p-value= 0.05. Top 10 genes with smallest p-values are highlighted with the location text.

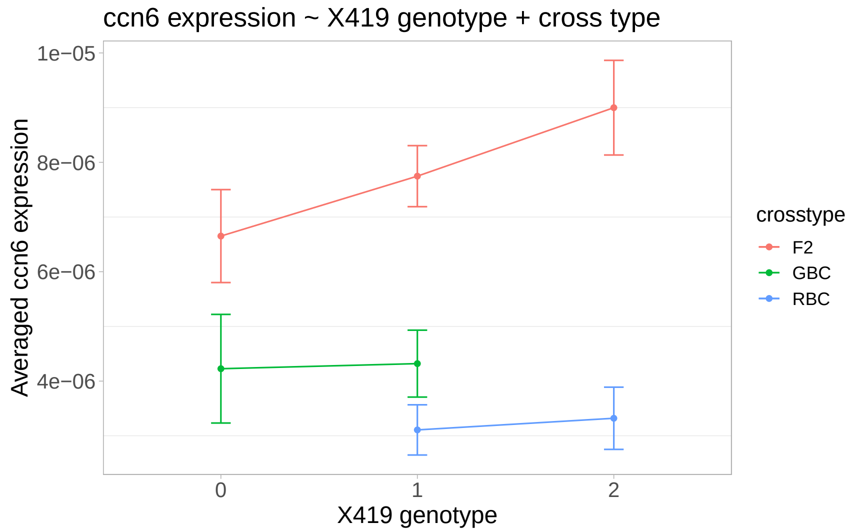

Figure S15: Averaged *ccn6* expressions under different X419 genotypes and cross types.

B.8 Stickleback analysis with different sparsity parameters

We also apply snQTL analysis with different sparsity parameter R to the stickleback data. First, we perform BIC selection for all gene 234 markers with candidate $c \in \{0.2, 0.3, 0.5, 0.7\}$ where $R = c^2 p$. Among 234 markers, 15 markers select $c = 0.2$, 51 markers choose $c = 0.3$, 82 markers choose $c = 0.5$ and 86 markers choose $c = 0.7$. Around 2/3 of markers fit the sparsity assumption in which only a few proportion of genes contribute to the joint network difference. This observation also indicates the necessity to introduce sparsity in the low-rank approximation. Second, we re-run the testing for the key snQTLs with different c's. Table S5 indicates that our snQTL detection results for stickleback data are robust to the sparsity parameters. Further, we check the leverage and joint differential network estimation at X419 with different c's. The list of top genes with highest leverages and the joint differential network remain the same as the main text results with $c = 0.3$, except slight shifts in leverage values. Hence, we conclude that overall snQTL analyses for stickleback data are robust.

c	0.2	0.3	0.5	0.7
X77	0.008	0.010	0.010	0.010
X81	0.012	0.012	0.012	0.012
X419	0.002	0.008	0.008	0.008
X423	0.008	0.008	0.008	0.008
X238	0.020	0.018	0.018	0.018
X245	0.018	0.020	0.020	0.020

Table S5: Empirical p-values of top snQTLs obtained by snQTL testing with different sparsity levels. We use $c = 0.3$ for the main text results.

References

[1] Weber JN, Steinel NC, Peng F, Shim KC, Lohman BK, Fuess LE, et al. Evolutionary gain and loss of a pathological immune response to parasitism. Science. 2022;377(6611):1206–1211.

[2] Zhu L, Lei J, Devlin B, Roeder K. Testing high-dimensional covariance matrices, with

application to detecting schizophrenia risk genes. The Annals of Applied Statistics. 2017;11(3):1810.

[3] Witten DM, Tibshirani R, Hastie T. A penalized matrix decomposition, with applications to sparse principal components and canonical correlation analysis. Biostatistics. 2009;10(3):515–534.
